# Supplementary material for: START (STrAtegies for RelaTives) coping strategy for family carers of adults with dementia: qualitative study of participants’ views about the intervention
Source: BMJ Open. 2014 Jun 4;4(6):e005273. doi: 10.1136/bmjopen-2014-005273 (PMC4054623; doi:10.1136/bmjopen-2014-005273)
Supplement: Author's manuscript [file bmjopen-2014-005273.draft_revisions.pdf]

BMJ Open

# START (STrAtegies for RelaTives) coping strategy for family carers of adults with dementia: Qualitative study of participants' views about the intervention.


| | |
|---|---|
| Journal: | *BMJ Open* |
| Manuscript ID: | bmjopen-2014-005273 |
| Article Type: | Research |
| Date Submitted by the Author: | 17-Mar-2014 |



| | |
|---|---|
| Complete List of Authors: | Sommerlad, Andrew; University College London, Division of Psychiatry<br>Manela, Monica; University College London, Division of Psychiatry<br>Cooper, Claudia; University College London, Division of Psychiatry<br>Rapaport, Penny; University College London, Division of Psychiatry<br>Livingston, Gill; University College London, Division of Psychiatry |



| | |
|---|---|
| <b>Primary Subject Heading</b>: | Mental health |
| Secondary Subject Heading: | Qualitative research |
| Keywords: | Dementia < NEUROLOGY, QUALITATIVE RESEARCH, Old age psychiatry < PSYCHIATRY, Depression & mood disorders < PSYCHIATRY, Anxiety disorders < PSYCHIATRY, Delirium & cognitive disorders < PSYCHIATRY |


SCHOLARONE™
Manuscripts

For peer review only

# START (STrAtegies for RelaTives) coping strategy for family carers of adults with dementia: Qualitative study of participants' views about the intervention.

**Corresponding author**:

Dr Andrew Sommerlad. Division of Psychiatry, University College London, Charles Bell House, 67-73 Riding House Street, London, W1W 7EJ, UK.

Tel: 020 7679 9248.

Email: a.sommerlad@ucl.ac.uk

**Authors**

Andrew Sommerlad*, Monica Manela*, Claudia Cooper*, Penny Rapaport*, Gill Livingston*

AS – Clinical Training Fellow

MM – Research Fellow

CC – Clinical Senior Lecturer

PR – Principal Clinical Psychologist

GL – Professor of Older People's Mental Health

*Division of Psychiatry, University College London, Charles Bell House, 67-73 Riding House Street, London, W1W 7EJ, UK.

**Running Title**

Qualitative experience of START intervention

**Key words**

Dementia, Carers, Depression, Psychosocial intervention

**Word Count**

4655

For peer review only

# START (STrAtegies for RelaTives) coping strategy for family carers of adults with dementia: Qualitative study of participants' views about the intervention.


## Abstract

*Objectives*

To analyse the experience of individual family carers of people with dementia who received a manual-based coping strategy programme, (STrAtegies for RelaTives, START), demonstrated in a randomised-controlled trial to reduce affective symptoms.

*Design*

A qualitative study using self-completed questionnaires exploring the experience of the START intervention. Two researchers transcribed, coded and analysed completed questionnaires thematically.

*Setting*

Three mental health and one neurology dementia clinic in South East England

*Participants*

Participants were primary family carers of a patient diagnosed with dementia who provided support at least weekly to their relative. We invited those in the treatment group remaining in the START study at two years post-randomisation (n=132) to participate. 75 people, comprising a maximum variation sample, responded.

*Primary and secondary outcome measures*

(1) Important aspects of the therapy. (2) Continued use of the intervention after the end of the therapy. (3) Unhelpful aspects of the therapy and suggestions for improvement. (4) Appropriate time for intervention delivery.

*Results*

Carers identified several different components as important: relaxation techniques, education about dementia, strategies to help manage the person with dementia's behaviour, contact with the therapist and changing unhelpful thoughts. Two-thirds of participants were aware of continuing to use the intervention's techniques at two year follow-up. Few participants suggested changes to intervention content, but some wanted more sessions and others more family members involved. Most were happy with receiving the intervention shortly after diagnosis, although some relatives of people with moderate dementia thought it should have been delivered at an earlier stage.

*Conclusions*


For peer review only

Participants' varied responses about which aspects of START were helpful, suggest a multicomponent intervention is suited to the differing circumstances of dementia carers, providing a range of potentially helpful strategies. The continued use of the strategies two years after receiving the intervention could be a mechanism for the intervention remaining effective.

## Article Summary

Strengths and limitations of this study

- This is the first study to analyse dementia family carers' experiences of and opinions about a clinically-effective complex psychological intervention (START).
- We gained a maximum variation sample; the participants in our study covered the spectrum of socio-demographic and clinical characteristics of the broader group of individuals who had received the START intervention.
- The structured questionnaires were administered two years after study entry and response was obtained from 57% of carers we contacted.
- The use of written questionnaires disadvantaged those with lower literacy and meant we could not probe further. They removed the need to please an interviewer.
- Despite efforts to do so, we were unable to obtain views from carers who had withdrawn from the study, suggesting that we under-sampled those who disliked the intervention or found it unacceptable.

## Introduction

The number of people with dementia is increasing due to an expanding older population. There are an estimated 670,000 people in the United Kingdom acting as primary family carers for people with dementia, saving the state £8 billion per year.[1] Dementia carers show high levels of psychological distress, including depression and anxiety.[2] This increases the risk of care home admission for the person with dementia.[3]

Varying interventions have been proposed to support dementia carers, but few are evidence based. Cognitive behavioural therapy reduces carer burden and depression,[4] but it is usually delivered by clinical psychologists who remain a relatively limited resource because they are highly-trained and, as a corollary, more expensive. The UK national strategy for improving access to psychological therapies is a stepped care approach, where graduates supervised by clinical psychologists deliver less intensive therapy, allowing clinical psychologists to offer more high intensity interventions to those with more complex needs.[5]

The *Coping with Caregiving* complex psychological intervention was developed in the USA for groups of family carers. It reduced carer depression and anger and improved self-efficacy.[6] We adapted the programme for delivery within the UK National Health Service and evaluated it in the START

For peer review only - http://bmjopen.bmj.com/site/about/guidelines.xhtml

(Strategies for Relatives) study; a pragmatic randomised-controlled-trial (RCT). Affective symptoms and case-level depression decreased and quality of life increased in carers receiving the intervention compared to those receiving treatment as usual over an eight month follow-up period[7] and this was cost-effective.[8]

Complex interventions comprise numerous components, which may be independent or interdependent, and the 'active ingredient' is usually hard to determine.[9] Finding out why multi-component interventions work is important for implementation and might enable the intervention to be refined, tailored for specific groups or reduced in length with associated economic benefits. Researchers have sought to understand mechanisms of action of psychological therapies through exploration of mediators and moderators, such as self-efficacy and coping.[10, 11] This is useful but, in addition, participants often have views on which aspects of an intervention were valuable and asking them about this directly, as part of trial process evaluation, has proved useful in diverse interventions. These have included breast-feeding support interventions,[12] CBT self-management of IBS,[13] and maintaining healthy behaviour change.[14]

This approach has not, to our knowledge, been used previously to evaluate complex interventions for dementia carers. We qualitatively analysed dementia carers' experiences of taking part in START, a complex intervention. We aimed to explore which aspects of the therapy carers found helpful and unhelpful; carers' perspectives on the stage of the illness at which the programme should be delivered and how the intervention could be developed to better meet their needs.

## Methods

### *Setting and intervention*

The START study was a pragmatic multi-centre RCT evaluating the effect on dementia carers' affective symptoms of eight one-hour sessions of a manual-based coping intervention compared to usual treatment. The study protocol has been detailed elsewhere.[7] The intervention was delivered by psychology graduates without clinical qualifications, as a face-to-face, individual intervention at a location chosen by the carer, usually their home. The sessions consisted of psycho-education about dementia, carer stress and access to emotional support; exploration of behaviours or situations that the carer found difficult and potential management strategies; challenging unhelpful thoughts; relaxation techniques accompanied by CDs of relaxation exercises; communication skills; planning pleasurable activities; future planning and maintaining skills learnt. The carers were also given homework to complete and a manual of the intervention, in which to record their work. The participants kept the CD and manual to allow their continued use.

### *Participants*

Consenting participants were included in the main START trial if they identified themselves as the primary family carer of a patient diagnosed with dementia who provided support at least weekly to

For peer review only - http://bmjopen.bmj.com/site/about/guidelines.xhtml

their relative, who was not living in 24-hour care and referred to one of four different settings (three mental health services and a tertiary neurological service for dementia).

260 carers were randomised, of whom 173 participants were in the intervention group, allocated with a ratio of 2:1 (intervention: treatment as usual) to allow for potential therapist clustering effects in the trial intervention arm. Over the 24-month follow-up period, 41 carers from the intervention group withdrew or were lost to follow-up. We invited the remaining 132 participants to take part in this qualitative substudy.

*Data collection and procedure*

At the 24-month follow-up interview, researchers gave participants a questionnaire, a covering letter and stamped envelope addressed to the trial manager (rather than the researcher with whom they had previously had contact). In 17 cases, the questionnaire was completed during the research interview with the researcher, who had never been the carer's therapist.

The questionnaire was developed with the carers on the trial management and steering committees and consisted of a self-completed questionnaire comprising the following questions:

- Was there anything that you found particularly helpful?
- How have you used the intervention (support sessions, manual or CD) since it ended?
- Is there anything you would do differently?
- Is there anything you would add in?
- Looking back, do you feel that you took part in the intervention at the right time?

We subsequently sent all participants a transcript of their original response along with a freepost envelope, asking them whether it was representative of their true views and to make amendments if they wished. This method of quality control and validation allows participants to ensure that the transcript is what they intended to say. We also sent questionnaires to the participants who had previously withdrawn from the study asking the following questions:

- What did you think of the support sessions and manual?
- Whether you did or did not attend the support sessions, was there anything we should change to make it more useful to you?

We evaluated questionnaire responses alongside sociodemographic and clinical data, including time since diagnosis of dementia, carers' anxiety and depression – measured by the Hospital Anxiety and Depression Scale (HADS),[15] a self-rated scale which has been validated for use in a variety of settings – and the severity of patients' dementia – measured by the clinical dementia rating (CDR),[16] which grades the level of impairment related to dementia. This quantitative data was collected at baseline and 24 months in the original study.

For peer review only

*Analysis*

We transcribed returned questionnaires verbatim and used a thematic framework approach for analysis. Two researchers (AS and MM) independently read the transcripts and identified a framework of initial themes which referred to the main study objectives. The researchers then used the qualitative software package NVivo (QSR International Pty Ltd. Version 9, 2010) to code the transcripts according to these themes and jointly developed a thematic map with a hierarchy of themes and categories. We have anonymised all quotations, providing non-specific demographic information and do not think that any carer could be identified.

## Results

*Demographics*

We received completed questionnaires from 75 participants (57% of the 132 participants at 24 months). Tables 1 and 2 detail baseline demographic and clinical characteristics of the participants who received the START intervention who did and did not complete our questionnaire. These covered the demographic and clinical characteristics of the whole group who were included in the study and were broadly comparable although in the group who completed qualitative questionnaires, spouses or partners of patients were under-represented, and children of people with dementia over-represented; related to this, the mean age of responders was slightly lower in those completing questionnaires and we had fewer responses from retired people and those living with the patient.

We received only one response from a withdrawn participant; this individual completed the START programme but withdrew from the study before the 24 month follow-up interview. None of the participants who had initially returned a completed questionnaire made notable changes to their responses when invited to do so.

We have annotated quotes to describe the participants' role ('w' = wife, 'h' = husband', 'd' = daughter, 's' = son, 'n' = niece) and numbered participants in the order in which the quotes are used, the severity of dementia at baseline and the carer's total HADS score at baseline interview and 24 month follow-up (e.g., 'HADS 12 → 7' = HADS score of 12 at baseline and 7 at 24 month interview). The HADS score at 12 months has been provided for two participants who did not complete HADS at 24 months.

Important aspects of the therapy

Participants valued diverse elements of the intervention and these are summarised in figure 3. The relaxation CDs were most commonly cited as being useful during the period of therapy and beyond, and 22 participants told us they continued to use these and the taught relaxation techniques:


For peer review only - http://bmjopen.bmj.com/site/about/guidelines.xhtml

*"The CDs are very relaxing … still very much being used today."* (w1; very mild dementia; HADS 4 → 13)

*"Relaxation exercises helped before bedtime to clear the mind."* (d2; moderate dementia; HADS 14 → 10 [12 months])

18 participants suggested that understanding the condition in detail made it easier to cope with their relative's symptoms and some mentioned appreciating learning gradually about dementia:

*"NHS services gave a lot of information at diagnosis; too much negative info at once. I felt START was more supportive and gave smaller bits at a time."* (w3; mild young-onset dementia; HADS 19 → 8)

This knowledge allowed some participants to feel more prepared for the future and this, coupled with effective communication skills, enabled them to cope better as challenges emerged:

*"Some of the problems that I eventually had to face had been discussed, making me aware of them and able to care better."* (w4; very mild dementia; HADS 12 → 10)

*"When she was in hospital, doctors took her off medications. I learnt to be more assertive to talk to doctors and got medications put back on."* (s5; talking about Acetyl Cholinesterase Inhibitors; mild dementia; HADS 11 → 7)

Advice on coping with behaviour and communication was welcomed by 11 participants and was noted by some to have reduced their own distress:

*"The most important and useful message was to go along with whatever the Alzheimer's sufferer says, i.e. enter their World and don't attempt to correct obvious inconsistencies."* (s6; moderate dementia; HADS 7 → 5)

Sessions on carer stress, using a cognitive therapeutic approach to help carers understand their own emotional responses and reframe negative thoughts, were noted by five participants to have been of practical help while others were grateful for what they saw as a rare chance to explore to their own emotional state:

*"Changing unhelpful thoughts … it concentrated my thoughts on how I was managing my own reactions and trying to be understanding of my husband's illness."* (w7; mild young-onset dementia; HADS 14 → 14)

*"What was an added bonus was that it centred on me rather than my husband. Previously all attention and energy had been focused on them."* (w8; moderate dementia; HADS 8 → 11)

For peer review only

The interaction with the therapist was valued by 17 participants, for varied reasons. Some were grateful for the opportunity to share their concerns with a professional; others appreciated the personal attributes of their therapist, while others noted the empathetic approach of the therapist and the validation of their own feelings:

> *"I think I found the 'talking through' with a knowledgeable person the most helpful."* (d9; very mild dementia; HADS 15 → 23)

> *"Therapist was lovely, warm."* (w10; very mild dementia; HADS 16 → 16)

> *"I felt it OK to be angry, upset, made to feel less guilty."* (d11; very mild dementia; HADS 18 → 13)

Ten participants commented that the START intervention had a prolonged impact on their lives, either because it empowered them to seek help after the therapy or because they had continued to apply some of the techniques and attitudes to other situations and shared them with other people:

> *"I have since joined the Alzheimer's Society, joined a yoga group and occasionally see a cognitive behavioural therapist – all of which were a result of taking part in the START project."* (w7; mild young-onset dementia; HADS 14 → 14)

> *"I have used the methods consistently within my working environment and in offering constructive advice and support to friends dealing with stressful situations that arise within their daily lives."* (n12; mild dementia; HADS 25 → 13)

<u>Participants' engagement with the therapy</u>

50 (67%) of those who responded to the questionnaire said that they had continued to use the intervention since the end of the sessions.

> *"Sometimes I sit and go through my orange folder* [therapy manual] *and there is a peace and understanding that someone is there with me."* (w13; mild dementia; HADS 23 → 17)

Of those who said they had not, ten gave no reason, three said that they had forgotten the sessions and in two cases their relative had died during the study. Other stated reasons are described below.

Feeling too busy or tired to continue to engage with the therapy was a frequently cited reason for not continuing to use the intervention, with one participant, the daughter of a woman with Alzheimer's Disease, commenting that she had little time to put the strategies into action once the protected therapy time had finished:

For peer review only

*"I found it helpful while the sessions were in progress, but lost the allocated time when it was over."* (d14; mild dementia; HADS 7 → 5)

Another carer stopped using the intervention because they felt they needed the support and guidance of the therapist. Some respondents commented that they had felt that the intervention was not relevant to their particular situation, either because the dementia was not severe, the caring difficulties did not relate directly to the effect of dementia or because of the particular symptoms they encountered:

*"Not really had to use it as my mother is still at an early stage."* (s15; mild dementia; HADS 3 → 2)

*"Caring problems were mainly physical rather than psychological."* (s16; moderate dementia; HADS 9 → 12)

*"I felt it was aimed at living with someone who has Alzheimer's which did not apply to me."* (d17; very mild dementia; HADS 8 → 14)

Three carers commented that the experience of the START intervention had encouraged them to make use of other techniques:

*"Rather than using the CD, I went back to practising transcendental meditation again – so thank you for that."* (w8; moderate dementia; HADS 8 → 11)

Unhelpful aspects of therapy and potential improvements

11 (15%) respondents suggested changes to the START therapy. Some commented that the nature of the intervention did not fit in with their approach or personality:

*"Wasn't something I would do for myself."* (w10; very mild dementia; HADS 16 → 16)

Five participants said they would have liked more sessions, with some suggesting a gradual rather than abrupt end to the programme:

*"Knowing that there would be a follow-up might have kept it all fresher in my mind for longer and got me into a routine of it all better."* (d14; mild dementia; HADS 7 → 5)

In contrast two participants commented that the sessions had been too demanding on their time:


For peer review only - http://bmjopen.bmj.com/site/about/guidelines.xhtml
For peer review only

*"The sessions were too long and interrupted normal daily duties."* (w18; mild dementia; HADS 16 → 34)

Five participants suggested support from other carers through group sessions or attending existing voluntary organisations would have been helpful:

*"[The Alzheimer's Society café] could have been used as the basis of a carer's group which would be of both practical and emotional help."* (w19; mild dementia; HADS 16 → 20)

It was intended that support sessions would include only the therapist and carer but two participants suggested other family members could have been included so they too could share the strategies:

*"Probably add one or two members of family on this programme in case the appointed carer is not able to do the caring."* (w1; very mild dementia; HADS 4 → 13)

Although one participant commented that they found it difficult to find a private place for the sessions in the house they shared with the person with dementia, two other carers suggested that including the relative in one or more sessions would have been helpful so that the therapist could tailor the sessions more appropriately or so that the person with dementia can understand the carers strain:

*"It would have been nice if the therapist met my Dad … to have the therapist's viewpoint, to see for themselves."* (d2; moderate dementia; HADS 14 → 10 [12 months])

*"One session involving the care-recipient so they appreciate there are problems … and the effect their illness is having on spouse ... might help with their self-control."* (w10; very mild dementia; HADS 16 → 16)

Two participants felt that the START sessions should have been more explicit in their exploration of the dementia future problems and prognosis:

*"More discussion of the likely course of the illness."* (s20; mild dementia; HADS 17 → 14)

*"How to prepare for what lies ahead."* (h21; moderate dementia; HADS 9 → 26)

Although the CD of relaxation techniques was popular with many respondents, others did not like it:

For peer review only

*"I haven't used the CD – some of which I found really irritating!"* (w22; moderate dementia; HADS 22 → 24)

*"I found the male voices off-putting on the CD – prefer all female voices."* (w3; mild young-onset dementia; HADS 19 → 8)

## Appropriate time for delivery of intervention

Participants were largely recruited shortly after or at the time of dementia diagnosis. 61 carers (81%) judged that they had taken part in the START intervention at the 'right time':

*"I now feel I have all the tools before she gets worse."* (s23; very mild dementia; HADS 12 → 5)

Of those who thought the intervention should have been offered at another time, eight wanted it earlier and six later. About three quarters of carers looking after those with very mild dementia (CDR 0.5) thought the intervention was delivered at the right time, rising to over 80% of those with relatives with mild or moderate dementia (CDR 1-2). Those who wanted it later tended to have relatives with milder dementia than those asking for it earlier. Among carers who would rather have received the intervention earlier, the median time since they reported being told the diagnosis was 5.5 months, 4 months for those who were happy with the time of delivery and 1.5 months for those who would rather have received it later.

Respondents commented that earlier engagement with the START programme would have helped them improve their communication and thus care better or avoid making major decisions regarding social care without being equipped with the necessary knowledge of dementia:

*"I wish I knew more, well before her condition was diagnosed, as I feel that I would have been more understanding and giving to her."* (d24; mild dementia; HADS 5 → 6)

*"[START programme] should have started earlier before we found a live-in carer for my mother-in-law."* (d25; mild dementia; HADS 11 → 6 [12 months])

Those who felt that the intervention was delivered too early felt it would have helped them cope with their relative's later deterioration:

*"I feel it was a little early as further down the line, I find it so much harder to cope with my mother as her Alzheimer's has got worse."* (d17; very mild dementia; HADS 8 → 14)



For peer review only - http://bmjopen.bmj.com/site/about/guidelines.xhtml

For peer review only

## Discussion

This study is the first to qualitatively analyse dementia carers' experiences of a complex psychological intervention and thus help us to understand the mechanisms by which it is effective. We asked carers about their experiences of the therapy two years after the 8-12 week therapy began, so we were able to explore how it worked immediately and whether benefits were noted some time later.

Our main finding is that the study participants valued diverse components of the intervention. The most frequently cited aspects were relaxation techniques, education about dementia, interaction with the therapist, cognitive techniques for their own thoughts and feelings, specific strategies for behavioural management and communication techniques for the person with dementia as well as learning to seek and ask for help. Most said they continued to use techniques at two year follow-up.

The heterogeneity of responses suggests that there were no particular aspects that were commonly unnecessary or unhelpful, and that a benefit of the multicomponent nature of this therapy is that it provides a diverse menu of strategies to suit the differing circumstances of dementia carers – in terms of the relative with dementia's symptoms; carer knowledge; social situation and support from mental health and social services; preferences and coping strategies.

The HADS scores for each quoted participant allow speculation regarding the impact of their anxiety and depression levels on their mental state at the time of both the intervention and providing this feedback which is instructive in some cases. However, they are used in illustrative quotes of participants, and cannot be assumed to represent other participants holding a similar view of the intervention.

Some of the responses demonstrate that participants had incorrectly interpreted the intervention's messages – for example, that teaching people with dementia about their condition 'might help with their self-control' or that one should 'go along with whatever the Alzheimer's sufferer says' (the message was to avoid criticism) – reflecting the subjective experience of psychological interventions but the overall effect of the intervention was nonetheless positive. While some HADS scores went up eg the first quote from 'w1', she continued to use the relaxation techniques and felt this was beneficial; it may be that she would have felt worse without the intervention.

Suggestions about how to improve the intervention focussed on form rather than content – for example, shorter or more numerous sessions, with several carers suggesting a longer period of intervention with reduced frequency of sessions. These participants may have been experiencing higher level of psychological distress, in which case additional sessions for those who are most distressed at the end of therapy would be in line with the stepped care approach to access to psychological therapies. Alternatively, they may have benefitted most and so were reluctant to stop.

Family carers have previously reported that receiving information over a longer period helps when making decisions about care [17-19] and respondents welcomed the gradual accumulation of knowledge about dementia. Others made suggestions about broadening session attendance to include other family members or the person with dementia. While including more family members


For peer review only - http://bmjopen.bmj.com/site/about/guidelines.xhtml

could reduce the individual feeling of therapist attention, it could broaden the impact of the intervention, and family interventions have been found to be effective in other studies.[20] Perhaps the diverse content helped the START intervention to support carers with a broad range of needs, and a flexible approach to its delivery, in terms of who is present in sessions and how they are scheduled, could assist implementation.

The contact with a professional was welcomed by many participants, who valued the empathetic approach, knowledge and interpersonal skills of the therapists. We know, from analysis of the effect of clustering by therapist, that the clinical effectiveness of the therapy was not dependent on which therapist delivered the intervention [7] so this suggests that supervised psychology graduates can deliver this therapy while maintaining a personal approach. Some carers cited a cognitive therapeutic approach as helpful and this supports research findings that cognitive reframing may be an effective aspect of individualised multicomponent interventions.[21]

*Strengths and weaknesses*

To our knowledge, our qualitative analysis of participants' experience of a clinically-effective and cost-effective psychosocial intervention aimed at improving the mental health of dementia carers is the first study of this type. In order to maximise the validity of our findings, we aimed for and succeeded in gaining, a maximum variation sample of people who completed the intervention; the participants in our study covered the spectrum of socio-demographic and clinical characteristics of a broader group of individuals who received the intervention. However, the questionnaire respondents, compared to those who did not respond, tended to be children rather than spouses of people with dementia, younger, less likely to be married, more likely to be in employment rather than retired and less likely to be living with the person with dementia.

In addition to this, the respondents had reached a higher educational level than non-respondents. It may be that participants with lower literacy attainment would have had more difficulties in filling in the questionnaire. The written format also meant that we could not probe participants' answers. For example, 18 participants specified that they appreciated receiving information about dementia but we do not know the opinion of the remaining 57 participants about this. Using self-completed questionnaires, however, had the strength that the participants were free to express their views. The lack of changes after we offered participants a chance to revise their transcripts also suggests this. It also supports the idea that the START intervention had a long-lasting and consistent effect on participants; the initial questionnaire responses providing a snapshot of the participants' views but these remained constant.

There is probably some response bias, with those who valued and benefited from the therapy most or least and had the strongest feelings being more likely to respond. As we did not receive any responses from participants whose relative had severe dementia at the beginning of the intervention, we cannot make assumptions about the experience of the intervention for this group. Nonetheless, many of the respondents cared for people who progressed to severe dementia or died, so delivering the intervention early may mean that it continues to confer benefit as dementia


For peer review only - http://bmjopen.bmj.com/site/about/guidelines.xhtml

deteriorates. We have been unable to obtain views from most carers who withdrew from the study, which would suggest that we probably under-sampled those who disliked the intervention or found it unacceptable. Nonetheless a minority of respondents did criticise the therapy, suggesting that our strategies to minimise social desirability bias were successful.

*Clinical implications and future research*

This analysis indicates aspects of the START intervention which were helpful and suggests some possible reasons for lasting clinical efficacy. Participants took part in a structured and guided intervention and their comments indicated a range of opinions about which parts were helpful. It seems that, although some individuals did not like components of the intervention, most were able to identify helpful aspects and that, by working with graduate psychologists over a period of time, they made some longer term changes. Supporting the structured sessions with a manual and CD based intervention was valuable for the two-thirds of participants who continued to use them two years later and participants commented that they also felt more capable of seeking help for future changing circumstances.

Our analysis has implications for when best to offer the intervention to carers in order to maximise engagement and potential benefit. Responses suggest that most carers want the intervention to be delivered shortly after they had been told of a dementia diagnosis; this allows future social care planning and psychological preparation for the developing illness. Some carers for people with very mild dementia would have preferred to wait a few months following diagnosis. In clinical practice, having flexibility in terms of when people are offered the START intervention could increase the acceptability of the intervention.

Overall, we found that two-thirds of carers reported continued use of the intervention after two years. This indicates a possible mechanism for continuing efficacy after the intervention's end. Retaining a copy of the manual and CD provides opportunity to revisit valued aspects of the therapy and adapt caring approaches to current challenges, while signposting to relevant voluntary organisations has been shown to be helpful[22] and empowered the participants in our study to seek help elsewhere, providing help as circumstances changed. Our findings are in line with others that multicomponent interventions are necessary in complex conditions with multiple domain pathology and that a single active ingredient may be illusory.[23, 24]

**Funding statement**

This project was funded by the National Institute for Health Research Health Technology Assessment (HTA) programme (project No 08/14/06). The authors analysed results and prepared this manuscript independently of the funding body. The views and opinions expressed therein are those of the authors and do not necessarily reflect those of the HTA programme, National Institute for Health Research, National Health Service, or the Department of Health. The study was sponsored by University College London. Neither funders nor sponsors had a role in the study design and the



For peer review only - http://bmjopen.bmj.com/site/about/guidelines.xhtml

collection, analysis and interpretation of data and the writing of the article and the decision to submit it for publication. The researchers were independent from funders and sponsors.

## Competing interests statement

All authors have completed the ICMJE uniform disclosure form at www.icmje.org/coi_disclosure.pdf and declare: support from Health Technology Assessment for the submitted work; no financial relationships with any organisations that might have an interest in the submitted work in the previous three years; no other relationships or activities that could appear to have influenced the submitted work.

## Description of authors' roles

AS, PR, CC and GL contributed to the design of the study. MM facilitated the questionnaire distribution. AS and MM analysed the data for themes and agreed on a coding frame. All authors contributed substantially to conception and design, or analysis and interpretation of data, and to drafting the article or revising it critically for intellectual content.

## Acknowledgements

We thank Dolores Gallagher Thompson for her original manual and allowing us to adapt it; the participating carers; the Camden and Islington NHS Foundation Trust, University College London Hospital, the North East London Foundation Trust, and the North Essex Partnership Foundation; Vincent Kirchner and Lisa Gee for referring many patients; members of the steering committee and the data monitoring committee. The START research team acknowledges the support of the National Institute for Health Research, through the Dementia and Neurodegenerative Research Network (DeNDRoN).

For peer review only

## References

1 Alzheimer's Society. (2012). Dementia 2012: A national challenge.

2 Mahoney R, Regan C, Katona C, et al. Anxiety and depression in family caregivers of people with Alzheimer Disease: the LASER-AD study. *Am J Geriatr Psychiatry* 2005;13(9):795-801. doi: 10.1097/00019442-200509000-00008

3 Gallagher D, Ni Mhaolain A, Crosby L, et al. Determinants of the desire to institutionalize in Alzheimer's caregivers. *Am J Alzheimers Dis Other Demen* 2011;26:205-211. doi: 10.1177/1533317511400307

4 Pinquart M, Sörensen S. Helping caregivers of persons with dementia: which interventions work and how large are their effects? *International Psychogeriatrics* 2006;18(4):577-95. doi: 10.1017/S1041610206003462

5 Talking therapies: a 4 year plan of action [Internet]. Department of Health; 2011. Available from: https://www.gov.uk/government/uploads/system/uploads/attachment_data/file/213765/dh_123985.pdf

6 Gallagher D, Thompson L, Silven D, et al. Problem solving for caregivers: Class leaders' manual. VA Palo Alto Health Care System and Stanford University, Palo Alto, CA. 1985.

7 Livingston G, Barber J, Rapaport P, et al. Clinical effectiveness of a manual based coping strategy programme (START, STrAtegies for RelaTives) in promoting the mental health of carers of family members with dementia: pragmatic randomised controlled trial. *BMJ* 2013;347:f6276. doi: 10.1136/bmj.f6276

8 Knapp M,King D, Romeo R, et al. Cost effectiveness of a manual based coping strategy programme in promoting the mental health of family carers of people with dementia (the START (STrAtegies for RelaTives) study): a pragmatic randomised controlled trial. *BMJ* 2013;347:f6342. doi: http://dx.doi.org/10.1136/bmj.f6342

9 Wells M, Williams B, Treweek S, et al. Intervention description is not enough: evidence from an in-depth multiple case study on the untold role and impact of context in randomised controlled trials of seven complex interventions. *Trials* 2012;13:95. doi: 10.1186/1745-6215-13-95

10 Li R, Cooper C, Livingston G. Relationship of coping style to mood and anxiety disorders in dementia carers. *Curr Opin Psychiatry* 2014;27(1):52-6. doi: 10.1097/YCO.0000000000000020

11 Murphy R, Cooper Z, Hollon S, et al. How do psychological treatments work? Investigating mediators of change. *Behav Res Ther* 2009;47(1):1-5. doi: 10.1016/j.brat.2008.10.001

12 Hoddinott P, Craig L, MacLennan G, et al. Process evaluation for the Feeding Support Team (FEST) randomised controlled feasibility trial of proactive and reactive telephone support for breastfeeding


For peer review only - http://bmjopen.bmj.com/site/about/guidelines.xhtml

women living in disadvantaged areas. *BMJ Open* 2012;2:e001039. doi: 10.1136/bmjopen-2012-001039

13 Tonkin-Crine S, Bishop F, Ellis M, et al. Exploring patients' views of a cognitive behavioral therapy-based website for the self-management of irritable bowel syndrome symptoms. *J Med Internet Res* 2013;15(9):e190. doi: 10.2196/jmir.2672

14 Penn L, Moffatt S, White M. Participants' perspective on maintaining behaviour change: a qualitative study within the European Diabetes Prevention Study. *BMC Public Health* 2008;8:235. doi: 10.1186/1471-2458-8-235

15 Zigmond A, Snaith R. The hospital anxiety and depression scale. *Acta Psychiatr Scand* 1983;67:361-70. doi: 10.1111/j.1600-0447.1983.tb09716.x

16 Hughes C, Berg L, Danziger W, et al. A new clinical scale for the staging of dementia. *Br J Psychiatry* 1982;140:566-72. doi: 10.1192/bjp.140.6.566

17 Wald C, Fahy M, Walker Z, et al. What to tell dementia caregivers—the rule of threes. *Int J Geriatr Psychiatry* 2003;18:313-7. doi: 10.1002/gps.828

18 Hirschman K, Kapo J, Karlawish J. Why doesn’t a family member of a person with advanced dementia use a substituted judgment when making a decision for that person? *Am J Geriatr Psychiatry* 2006;14:659-67. doi: 10.1097/01.JGP.0000203179.94036.69

19 Livingston G, Leavey G, Manela M, et al. Making decisions for people with dementia who lack capacity: qualitative study of family carers in UK. *BMJ* 2010;341:c4184. doi: 10.1136/bmj.c4184

20 Marriott A, Donaldson C, Tarrier N, et al. Effectiveness of cognitive-behavioural family intervention in reducing the burden of care in carers of patients with Alzheimer's disease. *Br J Psychiatry* 2000;176:557-62. doi: 10.1192/bjp.176.6.557

21 Vernooij-Dassen M, Draskovic I, McCleery, et al. Cognitive reframing for carers of people with dementia. *Cochrane Database of Systematic Reviews* 2001;11:CD005318. doi: 10.1002/14651858.CD005318.pub2

22 Sörensen S, Duberstein P, Gill D, et al. Dementia care: mental health effects, intervention strategies and clinical implications. *Lancet Neurol* 2006;5:961-73. doi: 10.1016/S1474-4422(06)705993

23 Spijker A, Vernooij-Dassen M, Vasse E, et al. Effectiveness of nonpharmacological interventions in delaying the institutionalization of patients with dementia: A meta-analysis. *J Am Geriatr Soc* 2008;56:1116-1128. doi: 10.1111/j.1532-5415.2008.01705.x

24 Olazarán J, Reisberg B, Clare L, *et al*. Nonpharmacological therapies in Alzheimer's disease: a systematic review of efficacy. *Dement Geriatr Cogn Disord* 2010;30:161-178. doi: 10.1159/000316119



For peer review only - http://bmjopen.bmj.com/site/about/guidelines.xhtml

**Table 1.** Baseline carer characteristics of questionnaire respondents and non-respondents

| | | Respondents (n=75) Mean (SD) | Non-respondents (n=98) Mean (SD) |
|---|---|---|---|
| **Age** | | 59.3 (13.7) Range: 18 - 85 | 64.1 (15.1) Range: 19 - 88 |
| | | | |
| | **Characteristic** | **n (%) of respondents (n=75)** | **n (%) of non-respondents (n=98)** |
| **Gender** | Female | 49 (65.3) | 67 (68.4) |
| | | | |
| **Ethnicity** | White UK | 58 (78.4) | 67 (68.4) |
| | White other | 4 (5.4) | 12 (12.2) |
| | Black and minority ethnic | 12 (16.2) | 19 (19.4) |
| | Missing | 1 | 0 |
| | | | |
| **Marital status** | Married / common law | 42 (56.0) | 63 (64.3) |
| | | | |
| **Education** | No qualifications | 14 (18.7) | 31 (31.6) |
| | School level | 24 (32.0) | 27 (27.6) |
| | Further education | 23 (30.7) | 24 (24.5) |
| | Other | 14 (18.7) | 16 (16.3) |
| | | | |
| **Employment** | Full time | 17 (22.7) | 19 (19.4) |
| | Part time | 17 (22.7) | 10 (10.2) |
| | Retired | 29 (38.7) | 51 (52.0) |
| | Not working | 12 (16.0) | 18 (18.4) |
| | | | |
| **Relationship to patient** | Spouse / partner | 31 (41.3) | 47 (48.0) |
| | Child | 34 (45.3) | 37 (37.8) |
| | Other | 10 (13.3) | 14 (14.3) |
| | | | |
| **Living with patient?** | Yes | 44 (58.7) | 69 (70.4) |

For peer review only

**Table 2.** **Clinical characteristics of questionnaire respondents and non-respondents**

| | | Respondents (n=75) Median (SD) | Non-respondents(n=98) Median (SD) |
|---|---|---|---|
| | | | |
| **Number of months since initial diagnosis** | | 3.5 (19.8)<br>Range: 0 – 96 | 4.0 (17.3)<br>Range: 0 – 108 |
| | | | |
| | | **Respondents (n=75) Mean (SD)** | **Non-respondents(n=98) Mean (SD)** |
| | | | |
| **HADS Baseline** | | 13.6 (6.9) | 13.4 (7.7) |
| | | | |
| **HADS 24m** | | 14.2 (8.1) | 12.9 (8.3) |
| | | | |
| | **Characteristic** | **n (%) of respondents (n=75)** | **n (%) of non-respondents (n=98)** |
| | | | |
| **CDR BL** | Very mild | 15 (20.0) | 15 (15.3) |
| | Mild | 41 (54.7) | 50 (51.0) |
| | Moderate | 19 (25.3) | 29 (29.6) |
| | Severe | 0 | 2 (2.0) |
| | Missing | 0 | 2 (2.0) |
| | | | |
| **CDR 24m** | Mild | 21 (30.4) | 15 (31.3) |
| | Moderate | 26 (37.7) | 19 (39.6) |
| | Severe | 11 (15.9) | 6 (12.5) |
| | Care recipient died | 11 (15.9) | 8 (16.7) |
| | Missing | 6 | 9 |
| | Withdrawn | 0 | 41 |

**Legend: HADS: Hospital Anxiety and Depression score, CDR: Clinical Dementia Rating score**

For peer review only - http://bmjopen.bmj.com/site/about/guidelines.xhtml

**Figure 3.** **Aspects of START intervention which were frequently described as helpful by participants**

| |
|---|
| **Influences on carer attitude**<br>• Greater acceptance of diagnosis and situation<br>• More tolerant to person with dementia<br>• Validation of own feelings |
| **Specific components of START intervention**<br>• CD and relaxation exercises<br>• Behavioural and communication advice<br>• Education about dementia |
| **Interaction with therapist**<br>• Sharing concerns with an independent person<br>• Empathy from therapist<br>• Reframing of negative automatic thoughts |
| **Continued use of techniques and attitudes**<br>• Seeking similar approaches through voluntary organisations<br>• Sharing techniques with friends and relatives |

For peer review only

BMJ Open

# START (STrAtegies for RelaTives) coping strategy for family carers of adults with dementia: Qualitative study of participants' views about the intervention.

| | |
|---|---|
| Journal: | *BMJ Open* |
| Manuscript ID: | bmjopen-2014-005273.R1 |
| Article Type: | Research |
| Date Submitted by the Author: | 14-May-2014 |
| Complete List of Authors: | Sommerlad, Andrew; University College London, Division of Psychiatry<br>Manela, Monica; University College London, Division of Psychiatry<br>Cooper, Claudia; University College London, Division of Psychiatry<br>Rapaport, Penny; University College London, Division of Psychiatry<br>Livingston, Gill; University College London, Division of Psychiatry |
| <b>Primary Subject Heading</b>: | Mental health |
| Secondary Subject Heading: | Qualitative research |
| Keywords: | Dementia < NEUROLOGY, QUALITATIVE RESEARCH, Old age psychiatry < PSYCHIATRY, Depression & mood disorders < PSYCHIATRY, Anxiety disorders < PSYCHIATRY, Delirium & cognitive disorders < PSYCHIATRY |
| | |

SCHOLARONE™
Manuscripts

For peer review only

# START (STrAtegies for RelaTives) coping strategy for family carers of adults with dementia: Qualitative study of participants' views about the intervention.

**Corresponding author**:

Dr Andrew Sommerlad. Division of Psychiatry, University College London, Charles Bell House, 67-73 Riding House Street, London, W1W 7EJ, UK.

Tel: 020 7679 9248.

Email: a.sommerlad@ucl.ac.uk

**Authors**

Andrew Sommerlad*, Monica Manela*, Claudia Cooper*, Penny Rapaport*, Gill Livingston*

AS – Clinical Training Fellow

MM – Research Fellow

CC – Clinical Senior Lecturer

PR – Principal Clinical Psychologist

GL – Professor of Older People's Mental Health

*Division of Psychiatry, University College London, Charles Bell House, 67-73 Riding House Street, London, W1W 7EJ, UK.

**Running Title**

Qualitative experience of START intervention

**Key words**

Dementia, Carers, Depression, Psychosocial intervention

**Word Count**

4665



For peer review only - http://bmjopen.bmj.com/site/about/guidelines.xhtml

# START (STrAtegies for RelaTives) coping strategy for family carers of adults with dementia: Qualitative study of participants' views about the intervention.


## Abstract

*Objectives*

To analyse the experience of individual family carers of people with dementia who received a manual-based coping strategy programme, (STrAtegies for RelaTives, START), demonstrated in a randomised-controlled trial to reduce affective symptoms.

*Design*

A qualitative study using self-completed questionnaires exploring the experience of the START intervention. Two researchers transcribed, coded and analysed completed questionnaires thematically.

*Setting*

Three mental health and one neurology dementia clinic in South East England.

*Participants*

Participants were primary family carers of a patient diagnosed with dementia who provided support at least weekly to their relative. We invited those in the treatment group remaining in the START study at two years post-randomisation (n=132) to participate. 75 people, comprising a maximum variation sample, responded.

*Primary and secondary outcome measures*

(1) Important aspects of the therapy. (2) Continued use of the intervention after the end of the therapy. (3) Unhelpful aspects of the therapy and suggestions for improvement. (4) Appropriate time for intervention delivery.

*Results*

Carers identified several different components as important: relaxation techniques, education about dementia, strategies to help manage the person with dementia's behaviour, contact with the therapist and changing unhelpful thoughts. Two-thirds of participants were aware of continuing to use the intervention's techniques at two year follow-up. Few participants suggested changes to intervention content, but some wanted more sessions and others more family members involved. Most were happy with receiving the intervention shortly after diagnosis, although some relatives of people with moderate dementia thought it should have been delivered at an earlier stage.

*Conclusions*


For peer review only

Participants' varied responses about which aspects of START were helpful, suggest a multicomponent intervention is suited to the differing circumstances of dementia carers, providing a range of potentially helpful strategies. The continued use of the strategies two years after receiving the intervention could be a mechanism for the intervention remaining effective.

**Article Summary**

Strengths and limitations of this study

- This is the first study to analyse dementia family carers' experiences of and opinions about a clinically-effective complex psychological intervention (START).
- We gained a maximum variation sample; the participants in our study covered the spectrum of socio-demographic and clinical characteristics of the broader group of individuals who had received the START intervention.
- The structured questionnaires were administered two years after study entry and response was obtained from 57% of carers we contacted.
- The use of written questionnaires disadvantaged those with lower literacy and meant we could not probe further. They removed the need to please an interviewer.
- Despite efforts to do so, we were unable to obtain views from carers who had withdrawn from the study, suggesting that we under-sampled those who disliked the intervention or found it unacceptable.

For peer review only

## Introduction

The number of people with dementia is increasing due to an expanding older population. There are an estimated 670,000 people in the United Kingdom acting as primary family carers for people with dementia, saving the state £8 billion per year.[1] Dementia carers show high levels of psychological distress, including depression and anxiety.[2] This increases the risk of care home admission for the person with dementia.[3]

Varying interventions have been proposed to support dementia carers, but few are evidence based. Cognitive behavioural therapy reduces carer burden and depression,[4] but it is usually delivered by clinical psychologists who remain a relatively limited resource because they are highly-trained and, as a corollary, more expensive. The UK national strategy for improving access to psychological therapies is a stepped care approach, where graduates supervised by clinical psychologists deliver less intensive therapy, allowing clinical psychologists to offer more high intensity interventions to those with more complex needs.[5]

The *Coping with Caregiving* complex psychological intervention was developed in the USA for groups of family carers. It reduced carer depression and anger and improved self-efficacy.[6] We adapted the programme for delivery within the UK National Health Service and evaluated it in the START (Strategies for Relatives) study; a pragmatic randomised-controlled-trial (RCT). Affective symptoms and case-level depression decreased and quality of life increased in carers receiving the intervention compared to those receiving treatment as usual over an eight month follow-up period[7] and was cost-effective.[8]

Complex interventions comprise numerous components, which may be independent or interdependent, and the 'active ingredient' is usually hard to determine.[9] Finding out why multi-component interventions work is important for implementation and might enable the intervention to be refined, tailored for specific groups or reduced in length with associated economic benefits. Researchers have sought to understand mechanisms of action of psychological therapies through exploration of mediators and moderators, such as self-efficacy and coping.[10, 11] This is useful but, in addition, participants often have views on which aspects of an intervention were valuable and asking them about this directly, as part of trial process evaluation, has proved useful in diverse interventions. These have included breast-feeding support interventions,[12] CBT self-management of IBS,[13] and maintaining healthy behaviour change.[14]

This approach has not, to our knowledge, been used previously to evaluate complex interventions for dementia carers. We qualitatively analysed dementia carers' experiences of taking part in START, a complex intervention. We aimed to explore which aspects of the therapy carers found helpful and unhelpful; carers' perspectives on the stage of the illness at which the programme should be delivered and how the intervention could be developed to better meet their needs.

## Methods

*Setting and intervention*

For peer review only

The START study was a pragmatic multi-centre RCT evaluating the effect on dementia carers' affective symptoms of eight one-hour sessions of a manual-based coping intervention compared to usual treatment. The study protocol has been detailed elsewhere.[7] The intervention was delivered by psychology graduates without clinical qualifications, as a face-to-face, individual intervention at a location chosen by the carer, usually their home. The sessions consisted of psycho-education about dementia, carer stress and access to emotional support; exploration of behaviours or situations that the carer found difficult and potential management strategies; challenging unhelpful thoughts; relaxation techniques accompanied by CDs of relaxation exercises; communication skills; planning pleasurable activities; future planning and maintaining skills learnt. The carers were also given homework to complete and a manual of the intervention, in which to record their work. The participants kept the CD and manual to allow their continued use.

*Participants*

Consenting participants were included in the main START trial if they identified themselves as the primary family carer of a patient diagnosed with dementia who provided support at least weekly to their relative, who was not living in 24-hour care and referred to one of four different settings (three mental health services and a tertiary neurological service for dementia).

260 carers were randomised, of whom 173 participants were in the intervention group, allocated with a ratio of 2:1 (intervention: treatment as usual) to allow for potential therapist clustering effects in the trial intervention arm. Over the 24-month follow-up period, 41 carers from the intervention group withdrew or were lost to follow-up. We invited the remaining 132 participants to take part in this qualitative substudy.

*Data collection and procedure*

At the 24-month follow-up interview, researchers gave participants a questionnaire, a covering letter and stamped envelope addressed to the trial manager (rather than the researcher with whom they had previously had contact).

The questionnaire was developed with the carers on the trial management and steering committees and consisted of a self-completed questionnaire comprising the following questions:

- Was there anything that you found particularly helpful?
- How have you used the intervention (support sessions, manual or CD) since it ended?
- Is there anything you would do differently?
- Is there anything you would add in?
- Looking back, do you feel that you took part in the intervention at the right time?

We subsequently sent all participants a transcript of their original response along with a freepost envelope, asking them whether it was representative of their true views and to make amendments if

For peer review only

they wished. This method of quality control and validation allows participants to ensure that the transcript is what they intended to say. We also sent questionnaires to the participants who had previously withdrawn from the study asking the following questions:

- What did you think of the support sessions and manual?
- Whether you did or did not attend the support sessions, was there anything we should change to make it more useful to you?

We evaluated questionnaire responses alongside sociodemographic and clinical data, including time since diagnosis of dementia, carers' anxiety and depression – measured by the Hospital Anxiety and Depression Scale (HADS),[15] a self-rated scale which has been validated for use in a variety of settings – and the severity of patients' dementia – measured by the clinical dementia rating (CDR),[16] which grades the level of impairment related to dementia. This quantitative data was collected at baseline and 24 months in the original study.

*Analysis*

We transcribed returned questionnaires verbatim and used a thematic framework approach [17] for analysis. Two researchers (AS and MM) independently read the transcripts and identified a framework of initial themes which referred to the main study objectives. The researchers then used the qualitative software package NVivo (QSR International Pty Ltd. Version 9, 2010) to code the transcripts according to these themes and jointly developed a thematic map with a hierarchy of themes and categories. We have anonymised all quotations, providing non-specific demographic information and do not think that any carer could be identified.

## Results

*Demographics*

We received completed questionnaires from 75 participants (57% of the 132 participants at 24 months); 17 of these questionnaires were completed during the research interview with the researcher, who had never been the carer's therapist and the remaining questionnaires were sent by post to our research team. Tables 1 and 2 detail baseline demographic and clinical characteristics of the participants who received the START intervention who did and did not complete our questionnaire. Those who did complete a questionnaire covered the demographic and clinical characteristics of the whole group although spouses or partners of patients were under-represented, and children of people with dementia over-represented; related to this, the mean age of responders was slightly lower in those completing questionnaires and we had fewer responses from retired people and those living with the patient. Comparison using appropriate statistical analysis demonstrates that the lower age of the questionnaire respondents was statistically significant ($p=0.03$) but the differences in other demographic or clinical characteristics were not statistically significant.


For peer review only - http://bmjopen.bmj.com/site/about/guidelines.xhtml

We received only one response from a withdrawn participant; this individual completed the START programme but withdrew from the study before the 24 month follow-up interview. None of the participants who had initially returned a completed questionnaire made notable changes to their responses when invited to do so.

Participants' comments are detailed below and captured within four broad themes: important aspects of the therapy, participants' engagement with the therapy, unhelpful aspects of therapy and potential improvements and appropriate time for delivery of the intervention. Selected quotes are used here to illustrate important viewpoints. We have annotated quotes to describe the participants' role ('w' = wife, 'h' = husband', 'd' = daughter, 's' = son, 'n' = niece) and numbered participants in the order in which the quotes are used, the severity of dementia at baseline and the carer's total HADS score at baseline interview and 24 month follow-up (e.g., 'HADS 12 → 7' = HADS score of 12 at baseline and 7 at 24 month interview). The HADS score at 12 months has been provided for two participants who did not complete HADS at 24 months.

<u>Important aspects of the therapy</u>

Participants valued diverse elements of the intervention and these are summarised in figure 1. The relaxation CDs were most commonly cited as being useful during the period of therapy and beyond, and 22/75 participants told us they continued to use these and the taught relaxation techniques:

> *"The CDs are very relaxing … still very much being used today."* (w1; very mild dementia; HADS 4 → 13)
>
> *"Relaxation exercises helped before bedtime to clear the mind."* (d2; moderate dementia; HADS 14 → 10 [12 months])

18/75 participants suggested that understanding the condition in detail made it easier to cope with their relative's symptoms and some mentioned appreciating learning gradually about dementia:

> *"NHS services gave a lot of information at diagnosis; too much negative info at once. I felt START was more supportive and gave smaller bits at a time."* (w3; mild young-onset dementia; HADS 19 → 8)

This knowledge allowed some participants to feel more prepared for the future and this, coupled with effective communication skills, enabled them to cope better as challenges emerged:

> *"Some of the problems that I eventually had to face had been discussed, making me aware of them and able to care better."* (w4; very mild dementia; HADS 12 → 10)
>
> *"When she was in hospital, doctors took her off medications. I learnt to be more assertive to talk to doctors and got medications put back on."* (s5; talking about Acetyl Cholinesterase Inhibitors; mild dementia; HADS 11 → 7)

For peer review only

Advice on coping with behaviour and communication was cited by 11/75 participants as welcome and was noted by some to have reduced their own distress:

> *"The most important and useful message was to go along with whatever the Alzheimer's sufferer says, i.e. enter their World and don't attempt to correct obvious inconsistencies."* (s6; moderate dementia; HADS 7 → 5)

Sessions on carer stress, using a cognitive therapeutic approach to help carers understand their own emotional responses and reframe negative thoughts, were noted by 5/75 participants to have been of practical help; some were grateful for what they saw as a rare chance to explore to their own emotional state:

> *"Changing unhelpful thoughts … it concentrated my thoughts on how I was managing my own reactions and trying to be understanding of my husband's illness."* (w7; mild young-onset dementia; HADS 14 → 14)

> *"What was an added bonus was that it centred on me rather than my husband. Previously all attention and energy had been focused on them."* (w8; moderate dementia; HADS 8 → 11)

17/75 participants told us that they valued the interaction with the therapist, for varied reasons. Some were grateful for the opportunity to share their concerns with a professional; others appreciated the personal attributes of their therapist, while others noted the empathetic approach of the therapist and the validation of their own feelings:

> *"I think I found the 'talking through' with a knowledgeable person the most helpful."* (d9; very mild dementia; HADS 15 → 23)

> *"Therapist was lovely, warm."* (w10; very mild dementia; HADS 16 → 16)

> *"I felt it OK to be angry, upset, made to feel less guilty."* (d11; very mild dementia; HADS 18 → 13)

10/75 participants commented that the START intervention had a prolonged impact on their lives, either because it empowered them to seek help after the therapy or because they had continued to apply some of the techniques and attitudes to other situations and shared them with other people:

> *"I have since joined the Alzheimer's Society, joined a yoga group and occasionally see a cognitive behavioural therapist – all of which were a result of taking part in the START project."* (w7; mild young-onset dementia; HADS 14 → 14)



For peer review only - http://bmjopen.bmj.com/site/about/guidelines.xhtml

For peer review only

*"I have used the methods consistently within my working environment and in offering constructive advice and support to friends dealing with stressful situations that arise within their daily lives."* (n12; mild dementia; HADS 25 → 13)

Participants' engagement with the therapy

50/75 of those who responded to the questionnaire said that they had continued to use the intervention since the end of the sessions.

*"Sometimes I sit and go through my orange folder* [therapy manual] *and there is a peace and understanding that someone is there with me."* (w13; mild dementia; HADS 23 → 17)

Of those who said they had not, ten gave no reason, three said that they had forgotten the sessions and in two cases their relative had died during the study. Other stated reasons are described below.

Feeling too busy or tired to continue to engage with the therapy was a frequently cited reason for not continuing to use the intervention, with one participant, the daughter of a woman with Alzheimer's Disease, commenting that she had little time to put the strategies into action once the protected therapy time had finished:

*"I found it helpful while the sessions were in progress, but lost the allocated time when it was over."* (d14; mild dementia; HADS 7 → 5)

Another carer stopped using the intervention because they felt they needed the support and guidance of the therapist. Some respondents commented that they had felt that the intervention was not relevant to their particular situation, either because the dementia was not severe, the caring difficulties did not relate directly to the effect of dementia or because of the particular symptoms they encountered:

*"Not really had to use it as my mother is still at an early stage."* (s15; mild dementia; HADS 3 → 2)

*"Caring problems were mainly physical rather than psychological."* (s16; moderate dementia; HADS 9 → 12)

*"I felt it was aimed at living with someone who has Alzheimer's which did not apply to me."* (d17; very mild dementia; HADS 8 → 14)

Three carers commented that the experience of the START intervention had encouraged them to make use of other techniques:

For peer review only

*"Rather than using the CD, I went back to practising transcendental meditation again – so thank you for that."* (w8; moderate dementia; HADS 8 → 11)

Unhelpful aspects of therapy and potential improvements

11/75 respondents suggested changes to the START therapy. Some commented that the nature of the intervention did not fit in with their approach or personality:

*"Wasn't something I would do for myself."* (w10; very mild dementia; HADS 16 → 16)

5/75 participants said they would have liked more sessions, with some suggesting a gradual rather than abrupt end to the programme:

*"Knowing that there would be a follow-up might have kept it all fresher in my mind for longer and got me into a routine of it all better."* (d14; mild dementia; HADS 7 → 5)

In contrast two participants commented that the sessions had been too demanding on their time:

*"The sessions were too long and interrupted normal daily duties."* (w18; mild dementia; HADS 16 → 34)

Five participants suggested support from other carers through group sessions or attending existing voluntary organisations would have been helpful:

*"[The Alzheimer's Society café] could have been used as the basis of a carer's group which would be of both practical and emotional help."* (w19; mild dementia; HADS 16 → 20)

It was intended that support sessions would include only the therapist and carer but two participants suggested other family members could have been included so they too could share the strategies:

*"Probably add one or two members of family on this programme in case the appointed carer is not able to do the caring."* (w1; very mild dementia; HADS 4 → 13)

Although one participant commented that they found it difficult to find a private place for the sessions in the house they shared with the person with dementia, two other carers suggested that


For peer review only - http://bmjopen.bmj.com/site/about/guidelines.xhtml

including the relative in one or more sessions would have been helpful so that the therapist could tailor the sessions more appropriately or so that the person with dementia can understand the carers strain:

> *"It would have been nice if the therapist met my Dad … to have the therapist's viewpoint, to see for themselves."* (d2; moderate dementia; HADS 14 → 10 [12 months])

> *"One session involving the care-recipient so they appreciate there are problems … and the effect their illness is having on spouse ... might help with their self-control."* (w10; very mild dementia; HADS 16 → 16)

Two participants stated that the START sessions should have been more explicit in their exploration of the dementia future problems and prognosis:

> *"More discussion of the likely course of the illness."* (s20; mild dementia; HADS 17 → 14)

> *"How to prepare for what lies ahead."* (h21; moderate dementia; HADS 9 → 26)

Although the CD of relaxation techniques was popular with many respondents, others did not like it:

> *"I haven't used the CD – some of which I found really irritating!"* (w22; moderate dementia; HADS 22 → 24)

> *"I found the male voices off-putting on the CD – prefer all female voices."* (w3; mild young-onset dementia; HADS 19 → 8)

<u>Appropriate time for delivery of intervention</u>

Participants were largely recruited shortly after or at the time of dementia diagnosis. 61/75 carers judged that they had taken part in the START intervention at the 'right time':

> *"I now feel I have all the tools before she gets worse."* (s23; very mild dementia; HADS 12 → 5)

Of those who thought the intervention should have been offered at another time, eight wanted it earlier and six later. About three quarters of carers looking after those with very mild dementia (CDR 0.5) thought the intervention was delivered at the right time, rising to over 80% of those with relatives with mild or moderate dementia (CDR 1-2). Those who wanted it later tended to have relatives with milder dementia than those asking for it earlier. Among carers who would rather have received the intervention earlier, the median time since they reported being told the diagnosis was

For peer review only - http://bmjopen.bmj.com/site/about/guidelines.xhtml

5.5 months, 4 months for those who were happy with the time of delivery and 1.5 months for those who would rather have received it later.

Respondents commented that earlier engagement with the START programme would have helped them improve their communication and thus care better or avoid making major decisions regarding social care without being equipped with the necessary knowledge of dementia:

> *"I wish I knew more, well before her condition was diagnosed, as I feel that I would have been more understanding and giving to her."* (d24; mild dementia; HADS 5 → 6)
>
> *"[START programme] should have started earlier before we found a live-in carer for my mother-in-law."* (d25; mild dementia; HADS 11 → 6 [12 months])

Those who felt that the intervention was delivered too early felt it would have helped them cope with their relative's later deterioration:

> *"I feel it was a little early as further down the line, I find it so much harder to cope with my mother as her Alzheimer's has got worse."* (d17; very mild dementia; HADS 8 → 14)

## Discussion

This study is the first to qualitatively analyse dementia carers' experiences of a complex psychological intervention and thus help us to understand the mechanisms by which it is effective. We asked carers about their experiences of the therapy two years after the 8-12 week therapy began, so we were able to explore how it worked immediately and whether benefits were noted some time later.

Our main finding is that the study participants valued diverse components of the intervention. The most frequently cited aspects were relaxation techniques, education about dementia, interaction with the therapist, cognitive techniques for their own thoughts and feelings, specific strategies for behavioural management and communication techniques for the person with dementia as well as learning to seek and ask for help. Most said they continued to use techniques at two year follow-up.

The heterogeneity of responses suggests that there were no particular aspects that were commonly unnecessary or unhelpful, and that a benefit of the multicomponent nature of this therapy is that it provides a diverse menu of strategies to suit the differing circumstances of dementia carers – in terms of the relative with dementia's symptoms; carer knowledge; social situation and support from mental health and social services; preferences and coping strategies.

The HADS scores for each quoted participant allow speculation regarding the impact of their anxiety and depression levels on their mental state at the time of both the intervention and providing this

For peer review only - http://bmjopen.bmj.com/site/about/guidelines.xhtml

feedback which is instructive in some cases. However, they are used in illustrative quotes of participants, and cannot be assumed to represent other participants holding a similar view of the intervention.

Some of the responses demonstrate that participants had incorrectly interpreted the intervention's messages – for example, that teaching people with dementia about their condition 'might help with their self-control' or that one should 'go along with whatever the Alzheimer's sufferer says' (the message was to avoid criticism) – reflecting the subjective experience of psychological interventions but the overall effect of the intervention was nonetheless positive. While some HADS scores went up eg the first quote from 'w1', she continued to use the relaxation techniques and felt this was beneficial; it may be that she would have felt worse without the intervention.

Suggestions about how to improve the intervention focussed on form rather than content – for example, shorter or more numerous sessions, with several carers suggesting a longer period of intervention with reduced frequency of sessions. These participants may have been experiencing higher level of psychological distress, in which case additional sessions for those who are most distressed at the end of therapy would be in line with the stepped care approach to access to psychological therapies. Alternatively, they may have benefitted most and so were reluctant to stop.

Family carers have previously reported that receiving information over a longer period helps when making decisions about care [18-20] and respondents welcomed the gradual accumulation of knowledge about dementia. Others made suggestions about broadening session attendance to include other family members or the person with dementia. While including more family members could reduce the individual feeling of therapist attention, it could broaden the impact of the intervention, and family interventions have been found to be effective in other studies.[21] Perhaps the diverse content helped the START intervention to support carers with a broad range of needs, and a flexible approach to its delivery, in terms of who is present in sessions and how they are scheduled, could assist implementation.

The contact with a professional was welcomed by many participants, who valued the empathetic approach, knowledge and interpersonal skills of the therapists. We know, from analysis of the effect of clustering by therapist, that the clinical effectiveness of the therapy was not dependent on which therapist delivered the intervention [7] so this suggests that supervised psychology graduates can deliver this therapy while maintaining a personal approach. Some carers cited a cognitive therapeutic approach as helpful and this supports research findings that cognitive reframing may be an effective aspect of individualised multicomponent interventions.[22]

*Strengths and weaknesses*

To our knowledge, our qualitative analysis of participants' experience of a clinically-effective and cost-effective psychosocial intervention aimed at improving the mental health of dementia carers is the first study of this type. In order to maximise the validity of our findings, we aimed for and succeeded in gaining, a maximum variation sample of people who completed the intervention; the


For peer review only - http://bmjopen.bmj.com/site/about/guidelines.xhtml

participants in our study covered the spectrum of socio-demographic and clinical characteristics of a broader group of individuals who received the intervention. However, the questionnaire respondents, compared to those who did not respond, were statistically significantly younger and tended to be children rather than spouses of people with dementia, less likely to be married, more likely to be in employment rather than retired and less likely to be living with the person with dementia.

In addition to this, the respondents had reached a higher educational level than non-respondents. It may be that participants with lower literacy attainment would have had more difficulties in filling in the questionnaire. The written format also meant that we could not probe participants' answers. For example, 18 participants specified that they appreciated receiving information about dementia but we do not know the opinion of the remaining 57 participants about this. Using self-completed questionnaires, however, had the strength that the participants were free to express their views. The lack of changes after we offered participants a chance to revise their transcripts also suggests this. It also supports the idea that the START intervention had a long-lasting and consistent effect on participants; the initial questionnaire responses providing a snapshot of the participants' views but these remained constant.

There is probably some response bias, with those who valued and benefited from the therapy most or least and had the strongest feelings being more likely to respond. As we did not receive any responses from participants whose relative had severe dementia at the beginning of the intervention, we cannot make assumptions about the experience of the intervention for this group. Nonetheless, many of the respondents cared for people who progressed to severe dementia or died, so delivering the intervention early may mean that it continues to confer benefit as dementia deteriorates. We have been unable to obtain views from most carers who withdrew from the study, which would suggest that we probably under-sampled those who disliked the intervention or found it unacceptable. Nonetheless a minority of respondents did criticise the therapy, suggesting that our strategies to minimise social desirability bias were successful.

*Clinical implications and future research*

This analysis indicates aspects of the START intervention which were helpful and suggests some possible reasons for lasting clinical efficacy.[23] Participants took part in a structured and guided intervention and their comments indicated a range of opinions about which parts were helpful. It seems that, although some individuals did not like components of the intervention, most were able to identify helpful aspects and that, by working with graduate psychologists over a period of time, they made some longer term changes. Supporting the structured sessions with a manual and CD based intervention was valuable for the two-thirds of participants who continued to use them two years later and participants commented that they also felt more capable of seeking help for future changing circumstances.

Our analysis has implications for when best to offer the intervention to carers in order to maximise engagement and potential benefit. Responses suggest that most carers want the intervention to be

For peer review only

delivered shortly after they had been told of a dementia diagnosis; this allows future social care planning and psychological preparation for the developing illness. Some carers for people with very mild dementia would have preferred to wait a few months following diagnosis. In clinical practice, having flexibility in terms of when people are offered the START intervention could increase the acceptability of the intervention.

Overall, we found that two-thirds of carers reported continued use of the intervention after two years. This indicates a possible mechanism for continuing efficacy after the intervention's end. Retaining a copy of the manual and CD provides opportunity to revisit valued aspects of the therapy and adapt caring approaches to current challenges, while signposting to relevant voluntary organisations has been shown to be helpful[24] and empowered the participants in our study to seek help elsewhere, providing help as circumstances changed. Our findings are in line with others that multicomponent interventions are necessary in complex conditions with multiple domain pathology and that a single active ingredient may be illusory.[25, 26]

For peer review only

**Acknowledgements**

We thank Dolores Gallagher Thompson for her original manual and allowing us to adapt it; the participating carers; the Camden and Islington NHS Foundation Trust, University College London Hospital, the North East London Foundation Trust, and the North Essex Partnership Foundation; Vincent Kirchner and Lisa Gee for referring many patients; members of the steering committee and the data monitoring committee. The START research team acknowledges the support of the National Institute for Health Research, through the Dementia and Neurodegenerative Research Network (DeNDRoN).

**Funding statement**

This project was funded by the National Institute for Health Research Health Technology Assessment (HTA) programme (project No 08/14/06). The authors analysed results and prepared this manuscript independently of the funding body. The views and opinions expressed therein are those of the authors and do not necessarily reflect those of the HTA programme, National Institute for Health Research, National Health Service, or the Department of Health. The study was sponsored by University College London. Neither funders nor sponsors had a role in the study design and the collection, analysis and interpretation of data and the writing of the article and the decision to submit it for publication. The researchers were independent from funders and sponsors.

**Description of authors' roles**

AS, PR, CC and GL contributed to the design of the study. MM facilitated the questionnaire distribution. AS and MM analysed the data for themes and agreed on a coding frame. All authors contributed substantially to conception and design, or analysis and interpretation of data, and to drafting the article or revising it critically for intellectual content.

**Competing interests statement**

All authors have completed the ICMJE uniform disclosure form at www.icmje.org/coi_disclosure.pdf and declare: support from Health Technology Assessment for the submitted work; no financial relationships with any organisations that might have an interest in the submitted work in the previous three years; no other relationships or activities that could appear to have influenced the submitted work.

**Data Sharing Statement**

No additional data available

**Ethical approval**

Ethical approval: The trial was conducted in accordance with Good Clinical Practice guidelines, the Declaration of Helsinki, the Clinical Trials Regulations and local laws and regulations. We obtained written ethics approval for the study from East London and the City Research Ethics Committee for

For peer review only

the trial (ID: 09\H0703\84) and Research and Development permission from the local trusts. All participants gave written informed consent.

**Figure 1.** **Aspects of START intervention which were frequently described as helpful by participants**

For peer review only

## References

1 Alzheimer's Society. (2012). Dementia 2012: A national challenge.

2 Mahoney R, Regan C, Katona C, et al. Anxiety and depression in family caregivers of people with Alzheimer Disease: the LASER-AD study. *Am J Geriatr Psychiatry* 2005;13(9):795-801. doi: 10.1097/00019442-200509000-00008

3 Gallagher D, Ni Mhaolain A, Crosby L, et al. Determinants of the desire to institutionalize in Alzheimer's caregivers. *Am J Alzheimers Dis Other Demen* 2011;26:205-211. doi: 10.1177/1533317511400307

4 Pinquart M, Sörensen S. Helping caregivers of persons with dementia: which interventions work and how large are their effects? *International Psychogeriatrics* 2006;18(4):577-95. doi: 10.1017/S1041610206003462

5 Talking therapies: a 4 year plan of action [Internet]. Department of Health; 2011. Available from: https://www.gov.uk/government/uploads/system/uploads/attachment_data/file/213765/dh_123985.pdf

6 Gallagher D, Thompson L, Silven D, et al. Problem solving for caregivers: Class leaders' manual. VA Palo Alto Health Care System and Stanford University, Palo Alto, CA. 1985.

7 Livingston G, Barber J, Rapaport P, et al. Clinical effectiveness of a manual based coping strategy programme (START, STrAtegies for RelaTives) in promoting the mental health of carers of family members with dementia: pragmatic randomised controlled trial. *BMJ* 2013;347:f6276. doi: 10.1136/bmj.f6276

8 Knapp M,King D, Romeo R, et al. Cost effectiveness of a manual based coping strategy programme in promoting the mental health of family carers of people with dementia (the START (STrAtegies for RelaTives) study): a pragmatic randomised controlled trial. *BMJ* 2013;347:f6342. doi: http://dx.doi.org/10.1136/bmj.f6342

9 Wells M, Williams B, Treweek S, et al. Intervention description is not enough: evidence from an in-depth multiple case study on the untold role and impact of context in randomised controlled trials of seven complex interventions. *Trials* 2012;13:95. doi: 10.1186/1745-6215-13-95

10 Li R, Cooper C, Livingston G. Relationship of coping style to mood and anxiety disorders in dementia carers. *Curr Opin Psychiatry* 2014;27(1):52-6. doi: 10.1097/YCO.0000000000000020

11 Murphy R, Cooper Z, Hollon S, et al. How do psychological treatments work? Investigating mediators of change. *Behav Res Ther* 2009;47(1):1-5. doi: 10.1016/j.brat.2008.10.001

12 Hoddinott P, Craig L, MacLennan G, et al. Process evaluation for the Feeding Support Team (FEST) randomised controlled feasibility trial of proactive and reactive telephone support for breastfeeding women living in disadvantaged areas. *BMJ Open* 2012;2:e001039. doi: 10.1136/bmjopen-2012-001039

13 Tonkin-Crine S, Bishop F, Ellis M, et al. Exploring patients' views of a cognitive behavioral therapy-based website for the self-management of irritable bowel syndrome symptoms. *J Med Internet Res* 2013;15(9):e190. doi: 10.2196/jmir.2672

14 Penn L, Moffatt S, White M. Participants' perspective on maintaining behaviour change: a qualitative study within the European Diabetes Prevention Study. *BMC Public Health* 2008;8:235. doi: 10.1186/1471-2458-8-235

15 Zigmond A, Snaith R. The hospital anxiety and depression scale. *Acta Psychiatr Scand* 1983;67:361-70. doi: 10.1111/j.1600-0447.1983.tb09716.x

16 Hughes C, Berg L, Danziger W, et al. A new clinical scale for the staging of dementia. *Br J Psychiatry* 1982;140:566-72. doi: 10.1192/bjp.140.6.566

17 Richie J, Spencer L. Qualitative data analysis for applied policy research. In: Bryman A, Burgess R, editors. Analysing qualitative data. London:Routledge; 1994. P. 173–194

For peer review only

18 Wald C, Fahy M, Walker Z, et al. What to tell dementia caregivers—the rule of threes. *Int J Geriatr Psychiatry* 2003;18:313-7. doi: 10.1002/gps.828

19 Hirschman K, Kapo J, Karlawish J. Why doesn't a family member of a person with advanced dementia use a substituted judgment when making a decision for that person? *Am J Geriatr Psychiatry* 2006;14:659-67. doi: 10.1097/01.JGP.0000203179.94036.69

20 Livingston G, Leavey G, Manela M, et al. Making decisions for people with dementia who lack capacity: qualitative study of family carers in UK. *BMJ* 2010;341:c4184. doi: 10.1136/bmj.c4184

21 Marriott A, Donaldson C, Tarrier N, et al. Effectiveness of cognitive-behavioural family intervention in reducing the burden of care in carers of patients with Alzheimer's disease. *Br J Psychiatry* 2000;176:557-62. doi: 10.1192/bjp.176.6.557

22 Vernooij-Dassen M, Draskovic I, McCleery, et al. Cognitive reframing for carers of people with dementia. *Cochrane Database of Systematic Reviews* 2001;11:CD005318. doi: 10.1002/14651858.CD005318.pub2

23 Livingston G, Barber J, Rapaport P, et al. START (STrAtegies for RelaTives) study: A pragmatic randomised controlled trial to determine the efficacy and cost effectiveness of a manual based coping strategy programme in promoting the mental health of carers of people with dementia. *Health Technol Assess*. Forthcoming 2014.

24 Sörensen S, Duberstein P, Gill D, et al. Dementia care: mental health effects, intervention strategies and clinical implications. *Lancet Neurol* 2006;5:961-73. doi: 10.1016/S1474-4422(06)705993

25 Spijker A, Vernooij-Dassen M, Vasse E, et al. Effectiveness of nonpharmacological interventions in delaying the institutionalization of patients with dementia: A meta-analysis. *J Am Geriatr Soc* 2008;56:1116-1128. doi: 10.1111/j.1532-5415.2008.01705.x

26 Olazarán J, Reisberg B, Clare L, *et al*. Nonpharmacological therapies in Alzheimer's disease: a systematic review of efficacy. *Dement Geriatr Cogn Disord* 2010;30:161-178. doi: 10.1159/000316119

For peer review only



For peer review only - http://bmjopen.bmj.com/site/about/guidelines.xhtml

**Table 1.** Baseline carer characteristics of questionnaire respondents and non-respondents

| | | Respondents (n=75) Mean (SD) | Non-respondents (n=98) Mean (SD) |
|---|---|---|---|
| **Age** | | 59.3 (13.7) Range: 18 - 85 | 64.1 (15.1) Range: 19 - 88 |
| | | | |
| | **Characteristic** | **n (%) of respondents (n=75)** | **n (%) of non-respondents (n=98)** |
| **Gender** | Female | 49 (65.3) | 67 (68.4) |
| | | | |
| **Ethnicity** | White UK | 58 (78.4) | 67 (68.4) |
| | White other | 4 (5.4) | 12 (12.2) |
| | Black and minority ethnic | 12 (16.2) | 19 (19.4) |
| | Missing | 1 | 0 |
| | | | |
| **Marital status** | Married / common law | 42 (56.0) | 63 (64.3) |
| | | | |
| **Education** | No qualifications | 14 (18.7) | 31 (31.6) |
| | School level | 24 (32.0) | 27 (27.6) |
| | Further education | 23 (30.7) | 24 (24.5) |
| | Other | 14 (18.7) | 16 (16.3) |
| | | | |
| **Employment** | Full time | 17 (22.7) | 19 (19.4) |
| | Part time | 17 (22.7) | 10 (10.2) |
| | Retired | 29 (38.7) | 51 (52.0) |
| | Not working | 12 (16.0) | 18 (18.4) |
| | | | |
| **Relationship to patient** | Spouse / partner | 31 (41.3) | 47 (48.0) |
| | Child | 34 (45.3) | 37 (37.8) |
| | Other | 10 (13.3) | 14 (14.3) |
| | | | |
| **Living with patient?** | Yes | 44 (58.7) | 69 (70.4) |

For peer review only

**Table 2.** Clinical characteristics of questionnaire respondents and non-respondents

| | | Respondents (n=75) Median (SD) | Non-respondents(n=98) Median (SD) |
|---|---|---|---|
| **Number of months since initial diagnosis** | | 3.5 (19.8)<br>Range: 0 – 96 | 4.0 (17.3)<br>Range: 0 – 108 |
| | | **Respondents (n=75) Mean (SD)** | **Non-respondents(n=98) Mean (SD)** |
| **HADS Baseline** | | 13.6 (6.9) | 13.4 (7.7) |
| **HADS 24m** | | 14.2 (8.1) | 12.9 (8.3) |
| | **Characteristic** | **n (%) of respondents (n=75)** | **n (%) of non-respondents (n=98)** |
| **CDR BL** | Very mild | 15 (20.0) | 15 (15.3) |
| | Mild | 41 (54.7) | 50 (51.0) |
| | Moderate | 19 (25.3) | 29 (29.6) |
| | Severe | 0 | 2 (2.0) |
| | Missing | 0 | 2 (2.0) |
| **CDR 24m** | Mild | 21 (30.4) | 15 (31.3) |
| | Moderate | 26 (37.7) | 19 (39.6) |
| | Severe | 11 (15.9) | 6 (12.5) |
| | Care recipient died | 11 (15.9) | 8 (16.7) |
| | Missing | 6 | 9 |
| | Withdrawn | 0 | 41 |

**Legend: HADS: Hospital Anxiety and Depression score, CDR: Clinical Dementia Rating score**

For peer review only

**START (STrAtegies for RelaTives) coping strategy for family carers of adults with dementia: Qualitative study of participants' views about the intervention.**

**Corresponding author**:

Dr Andrew Sommerlad. Division of Psychiatry, University College London, Charles Bell House, 67-73 Riding House Street, London, W1W 7EJ, UK.

Tel: 020 7679 9248.

Email: a.sommerlad@ucl.ac.uk

**Authors**

Andrew Sommerlad*, Monica Manela*, Claudia Cooper*, Penny Rapaport*, Gill Livingston*

AS – Clinical Training Fellow

MM – Research Fellow

CC – Clinical Senior Lecturer

PR – Principal Clinical Psychologist

GL – Professor of Older People's Mental Health

*Division of Psychiatry, University College London, Charles Bell House, 67-73 Riding House Street, London, W1W 7EJ, UK.

**Running Title**

Qualitative experience of START intervention

**Key words**

Dementia, Carers, Depression, Psychosocial intervention

**Word Count**

~~4655~~4665



For peer review only

**START (STrAtegies for RelaTives) coping strategy for family carers of adults with dementia: Qualitative study of participants' views about the intervention.**


## Abstract

*Objectives*

To analyse the experience of individual family carers of people with dementia who received a manual-based coping strategy programme, (STrAtegies for RelaTives, START), demonstrated in a randomised-controlled trial to reduce affective symptoms.

*Design*

A qualitative study using self-completed questionnaires exploring the experience of the START intervention. Two researchers transcribed, coded and analysed completed questionnaires thematically.

*Setting*

Three mental health and one neurology dementia clinic in South East England.

*Participants*

Participants were primary family carers of a patient diagnosed with dementia who provided support at least weekly to their relative. We invited those in the treatment group remaining in the START study at two years post-randomisation (n=132) to participate. 75 people, comprising a maximum variation sample, responded.

*Primary and secondary outcome measures*

(1) Important aspects of the therapy. (2) Continued use of the intervention after the end of the therapy. (3) Unhelpful aspects of the therapy and suggestions for improvement. (4) Appropriate time for intervention delivery.

*Results*

Carers identified several different components as important: relaxation techniques, education about dementia, strategies to help manage the person with dementia's behaviour, contact with the therapist and changing unhelpful thoughts. Two-thirds of participants were aware of continuing to use the intervention's techniques at two year follow-up. Few participants suggested changes to intervention content, but some wanted more sessions and others more family members involved. Most were happy with receiving the intervention shortly after diagnosis, although some relatives of people with moderate dementia thought it should have been delivered at an earlier stage.

*Conclusions*




For peer review only - http://bmjopen.bmj.com/site/about/guidelines.xhtml

Participants' varied responses about which aspects of START were helpful, suggest a multicomponent intervention is suited to the differing circumstances of dementia carers, providing a range of potentially helpful strategies. The continued use of the strategies two years after receiving the intervention could be a mechanism for the intervention remaining effective.

**Article Summary**

Strengths and limitations of this study

- This is the first study to analyse dementia family carers' experiences of and opinions about a clinically-effective complex psychological intervention (START).
- We gained a maximum variation sample; the participants in our study covered the spectrum of socio-demographic and clinical characteristics of the broader group of individuals who had received the START intervention.
- The structured questionnaires were administered two years after study entry and response was obtained from 57% of carers we contacted.
- The use of written questionnaires disadvantaged those with lower literacy and meant we could not probe further. They removed the need to please an interviewer.
- Despite efforts to do so, we were unable to obtain views from carers who had withdrawn from the study, suggesting that we under-sampled those who disliked the intervention or found it unacceptable.

**Introduction**

The number of people with dementia is increasing due to an expanding older population. There are an estimated 670,000 people in the United Kingdom acting as primary family carers for people with dementia, saving the state £8 billion per year.[1] Dementia carers show high levels of psychological distress, including depression and anxiety.[2] This increases the risk of care home admission for the person with dementia.[3]

Varying interventions have been proposed to support dementia carers, but few are evidence based. Cognitive behavioural therapy reduces carer burden and depression,[4] but it is usually delivered by clinical psychologists who remain a relatively limited resource because they are highly-trained and, as a corollary, more expensive. The UK national strategy for improving access to psychological therapies is a stepped care approach, where graduates supervised by clinical psychologists deliver less intensive therapy, allowing clinical psychologists to offer more high intensity interventions to those with more complex needs.[5]

The *Coping with Caregiving* complex psychological intervention was developed in the USA for groups of family carers. It reduced carer depression and anger and improved self-efficacy.[6] We adapted the programme for delivery within the UK National Health Service and evaluated it in the START



For peer review only

(Strategies for Relatives) study; a pragmatic randomised-controlled-trial (RCT). Affective symptoms and case-level depression decreased and quality of life increased in carers receiving the intervention compared to those receiving treatment as usual over an eight month follow-up period[7] and ~~this~~ was cost-effective.[8]

Complex interventions comprise numerous components, which may be independent or interdependent, and the 'active ingredient' is usually hard to determine.[9] Finding out why multi-component interventions work is important for implementation and might enable the intervention to be refined, tailored for specific groups or reduced in length with associated economic benefits. Researchers have sought to understand mechanisms of action of psychological therapies through exploration of mediators and moderators, such as self-efficacy and coping.[10, 11] This is useful but, in addition, participants often have views on which aspects of an intervention were valuable and asking them about this directly, as part of trial process evaluation, has proved useful in diverse interventions. These have included breast-feeding support interventions,[12] CBT self-management of IBS,[13] and maintaining healthy behaviour change.[14]

This approach has not, to our knowledge, been used previously to evaluate complex interventions for dementia carers. We qualitatively analysed dementia carers' experiences of taking part in START, a complex intervention. We aimed to explore which aspects of the therapy carers found helpful and unhelpful; carers' perspectives on the stage of the illness at which the programme should be delivered and how the intervention could be developed to better meet their needs.

## Methods

*Setting and intervention*

The START study was a pragmatic multi-centre RCT evaluating the effect on dementia carers' affective symptoms of eight one-hour sessions of a manual-based coping intervention compared to usual treatment. The study protocol has been detailed elsewhere.[7] The intervention was delivered by psychology graduates without clinical qualifications, as a face-to-face, individual intervention at a location chosen by the carer, usually their home. The sessions consisted of psycho-education about dementia, carer stress and access to emotional support; exploration of behaviours or situations that the carer found difficult and potential management strategies; challenging unhelpful thoughts; relaxation techniques accompanied by CDs of relaxation exercises; communication skills; planning pleasurable activities; future planning and maintaining skills learnt. The carers were also given homework to complete and a manual of the intervention, in which to record their work. The participants kept the CD and manual to allow their continued use.

*Participants*

Consenting participants were included in the main START trial if they identified themselves as the primary family carer of a patient diagnosed with dementia who provided support at least weekly to



For peer review only - http://bmjopen.bmj.com/site/about/guidelines.xhtml

their relative, who was not living in 24-hour care and referred to one of four different settings (three mental health services and a tertiary neurological service for dementia).

260 carers were randomised, of whom 173 participants were in the intervention group, allocated with a ratio of 2:1 (intervention: treatment as usual) to allow for potential therapist clustering effects in the trial intervention arm. Over the 24-month follow-up period, 41 carers from the intervention group withdrew or were lost to follow-up. We invited the remaining 132 participants to take part in this qualitative substudy.

*Data collection and procedure*

At the 24-month follow-up interview, researchers gave participants a questionnaire, a covering letter and stamped envelope addressed to the trial manager (rather than the researcher with whom they had previously had contact). ~~In 17 cases, the questionnaire was completed during the research interview with the researcher, who had never been the carer's therapist.~~

The questionnaire was developed with the carers on the trial management and steering committees and consisted of a self-completed questionnaire comprising the following questions:

- Was there anything that you found particularly helpful?
- How have you used the intervention (support sessions, manual or CD) since it ended?
- Is there anything you would do differently?
- Is there anything you would add in?
- Looking back, do you feel that you took part in the intervention at the right time?

We subsequently sent all participants a transcript of their original response along with a freepost envelope, asking them whether it was representative of their true views and to make amendments if they wished. This method of quality control and validation allows participants to ensure that the transcript is what they intended to say. We also sent questionnaires to the participants who had previously withdrawn from the study asking the following questions:

- What did you think of the support sessions and manual?
- Whether you did or did not attend the support sessions, was there anything we should change to make it more useful to you?

We evaluated questionnaire responses alongside sociodemographic and clinical data, including time since diagnosis of dementia, carers' anxiety and depression – measured by the Hospital Anxiety and Depression Scale (HADS),[15] a self-rated scale which has been validated for use in a variety of settings – and the severity of patients' dementia – measured by the clinical dementia rating (CDR),[16] which grades the level of impairment related to dementia. This quantitative data was collected at baseline and 24 months in the original study.



For peer review only - http://bmjopen.bmj.com/site/about/guidelines.xhtml

*Analysis*

We transcribed returned questionnaires verbatim and used a thematic framework approach for analysis. Two researchers (AS and MM) independently read the transcripts and identified a framework of initial themes which referred to the main study objectives. The researchers then used the qualitative software package NVivo (QSR International Pty Ltd. Version 9, 2010) to code the transcripts according to these themes and jointly developed a thematic map with a hierarchy of themes and categories. We have anonymised all quotations, providing non-specific demographic information and do not think that any carer could be identified.

**Comment [ATS1]:** REFERENCE ADDED: Richie J, Spencer L. Qualitative data analysis for applied policy research. In: Bryman A, Burgess R, editors. Analysing qualitative data. London:Routledge; 1994. P. 173–194

## Results

*Demographics*

We received completed questionnaires from 75 participants (57% of the 132 participants at 24 months); 17 of these questionnaires were completed during the research interview with the researcher, who had never been the carer's therapist and the remaining questionnaires were sent by post to our research team.. Tables 1 and 2 detail baseline demographic and clinical characteristics of the participants who received the START intervention who did and did not complete our questionnaire. Those who did complete a questionnaire covered the demographic and clinical characteristics of the whole group although spouses or partners of patients were under-represented, and children of people with dementia over-represented; related to this, the mean age of responders was slightly lower in those completing questionnaires and we had fewer responses from retired people and those living with the patient. Comparison using appropriate statistical analysis demonstrates that the lower age of the questionnaire respondents was statistically significant (p=0.03) but the differences in other demographic or clinical characteristics were not statistically significant.~~These covered the demographic and clinical characteristics of the whole group who were included in the study and were broadly comparable although in the group who completed qualitative questionnaires, spouses or partners of patients were under-represented, and children of people with dementia over-represented; related to this, the mean age of responders was slightly lower in those completing questionnaires and we had fewer responses from retired people and those living with the patient.~~

We received only one response from a withdrawn participant; this individual completed the START programme but withdrew from the study before the 24 month follow-up interview. None of the participants who had initially returned a completed questionnaire made notable changes to their responses when invited to do so.

Participants' comments are detailed below and captured within four broad themes: important aspects of the therapy, participants' engagement with the therapy, unhelpful aspects of therapy and potential improvements and appropriate time for delivery of the intervention. Selected quotes are used here to illustrate important viewpoints. We have annotated quotes to describe the participants' role ('w' = wife, 'h' = husband', 'd' = daughter, 's' = son, 'n' = niece) and numbered participants in the



For peer review only

For peer review only - http://bmjopen.bmj.com/site/about/guidelines.xhtml

order in which the quotes are used, the severity of dementia at baseline and the carer's total HADS score at baseline interview and 24 month follow-up (e.g., 'HADS 12 → 7' = HADS score of 12 at baseline and 7 at 24 month interview). The HADS score at 12 months has been provided for two participants who did not complete HADS at 24 months.

Important aspects of the therapy

Participants valued diverse elements of the intervention and these are summarised in figure ~~3~~1. The relaxation CDs were most commonly cited as being useful during the period of therapy and beyond, and 22/75 participants told us they continued to use these and the taught relaxation techniques:

> *"The CDs are very relaxing … still very much being used today."* (w1; very mild dementia; HADS 4 → 13)

> *"Relaxation exercises helped before bedtime to clear the mind."* (d2; moderate dementia; HADS 14 → 10 [12 months])

18/75 participants suggested that understanding the condition in detail made it easier to cope with their relative's symptoms and some mentioned appreciating learning gradually about dementia:

> *"NHS services gave a lot of information at diagnosis; too much negative info at once. I felt START was more supportive and gave smaller bits at a time."* (w3; mild young-onset dementia; HADS 19 → 8)

This knowledge allowed some participants to feel more prepared for the future and this, coupled with effective communication skills, enabled them to cope better as challenges emerged:

> *"Some of the problems that I eventually had to face had been discussed, making me aware of them and able to care better."* (w4; very mild dementia; HADS 12 → 10)

> *"When she was in hospital, doctors took her off medications. I learnt to be more assertive to talk to doctors and got medications put back on."* (s5; talking about Acetyl Cholinesterase Inhibitors; mild dementia; HADS 11 → 7)

Advice on coping with behaviour and communication was was cited by 11/75 participants as welcome ~~welcomed by 11 participants~~ and was noted by some to have reduced their own distress:

> *"The most important and useful message was to go along with whatever the Alzheimer's sufferer says, i.e. enter their World and don't attempt to correct obvious inconsistencies."* (s6; moderate dementia; HADS 7 → 5)



For peer review only - http://bmjopen.bmj.com/site/about/guidelines.xhtml

Sessions on carer stress, using a cognitive therapeutic approach to help carers understand their own emotional responses and reframe negative thoughts, were noted by ~~five~~ 5/75 participants to have been of practical help while others were grateful for what they saw as a rare chance to explore to their own emotional state:

> *"Changing unhelpful thoughts … it concentrated my thoughts on how I was managing my own reactions and trying to be understanding of my husband's illness."* (w7; mild young-onset dementia; HADS 14 → 14)

> *"What was an added bonus was that it centred on me rather than my husband. Previously all attention and energy had been focused on them."* (w8; moderate dementia; HADS 8 → 11)

17/75 participants told us that they valued the interaction with the therapist~~The interaction with the therapist was valued by 17 participants~~, for varied reasons. Some were grateful for the opportunity to share their concerns with a professional; others appreciated the personal attributes of their therapist, while others noted the empathetic approach of the therapist and the validation of their own feelings:

> *"I think I found the 'talking through' with a knowledgeable person the most helpful."* (d9; very mild dementia; HADS 15 → 23)

> *"Therapist was lovely, warm."* (w10; very mild dementia; HADS 16 → 16)

> *"I felt it OK to be angry, upset, made to feel less guilty."* (d11; very mild dementia; HADS 18 → 13)

~~Ten~~ 10/75 participants commented that the START intervention had a prolonged impact on their lives, either because it empowered them to seek help after the therapy or because they had continued to apply some of the techniques and attitudes to other situations and shared them with other people:

> *"I have since joined the Alzheimer's Society, joined a yoga group and occasionally see a cognitive behavioural therapist – all of which were a result of taking part in the START project."* (w7; mild young-onset dementia; HADS 14 → 14)

> *"I have used the methods consistently within my working environment and in offering constructive advice and support to friends dealing with stressful situations that arise within their daily lives."* (n12; mild dementia; HADS 25 → 13)

Participants' engagement with the therapy



For peer review only

For peer review only - http://bmjopen.bmj.com/site/about/guidelines.xhtml

50/~~75 (67%)~~ of those who responded to the questionnaire said that they had continued to use the intervention since the end of the sessions.

> *"Sometimes I sit and go through my orange folder* [therapy manual] *and there is a peace and understanding that someone is there with me."* (w13; mild dementia; HADS 23 → 17)

Of those who said they had not, ten gave no reason, three said that they had forgotten the sessions and in two cases their relative had died during the study. Other stated reasons are described below.

Feeling too busy or tired to continue to engage with the therapy was a frequently cited reason for not continuing to use the intervention, with one participant, the daughter of a woman with Alzheimer's Disease, commenting that she had little time to put the strategies into action once the protected therapy time had finished:

> *"I found it helpful while the sessions were in progress, but lost the allocated time when it was over."* (d14; mild dementia; HADS 7 → 5)

Another carer stopped using the intervention because they felt they needed the support and guidance of the therapist. Some respondents commented that they had felt that the intervention was not relevant to their particular situation, either because the dementia was not severe, the caring difficulties did not relate directly to the effect of dementia or because of the particular symptoms they encountered:

> *"Not really had to use it as my mother is still at an early stage."* (s15; mild dementia; HADS 3 → 2)

> *"Caring problems were mainly physical rather than psychological."* (s16; moderate dementia; HADS 9 → 12)

> *"I felt it was aimed at living with someone who has Alzheimer's which did not apply to me."* (d17; very mild dementia; HADS 8 → 14)

Three carers commented that the experience of the START intervention had encouraged them to make use of other techniques:

> *"Rather than using the CD, I went back to practising transcendental meditation again – so thank you for that."* (w8; moderate dementia; HADS 8 → 11)

<u>Unhelpful aspects of therapy and potential improvements</u>



For peer review only - http://bmjopen.bmj.com/site/about/guidelines.xhtml

11/75 (15%) respondents suggested changes to the START therapy. Some commented that the nature of the intervention did not fit in with their approach or personality:

> *"Wasn't something I would do for myself."* (w10; very mild dementia; HADS 16 → 16)

Five 5/75 participants said they would have liked more sessions, with some suggesting a gradual rather than abrupt end to the programme:

> *"Knowing that there would be a follow-up might have kept it all fresher in my mind for longer and got me into a routine of it all better."* (d14; mild dementia; HADS 7 → 5)

In contrast two participants commented that the sessions had been too demanding on their time:

> *"The sessions were too long and interrupted normal daily duties."* (w18; mild dementia; HADS 16 → 34)

Five participants suggested support from other carers through group sessions or attending existing voluntary organisations would have been helpful:

> *"[The Alzheimer's Society café] could have been used as the basis of a carer's group which would be of both practical and emotional help."* (w19; mild dementia; HADS 16 → 20)

It was intended that support sessions would include only the therapist and carer but two participants suggested other family members could have been included so they too could share the strategies:

> *"Probably add one or two members of family on this programme in case the appointed carer is not able to do the caring."* (w1; very mild dementia; HADS 4 → 13)

Although one participant commented that they found it difficult to find a private place for the sessions in the house they shared with the person with dementia, two other carers suggested that including the relative in one or more sessions would have been helpful so that the therapist could tailor the sessions more appropriately or so that the person with dementia can understand the carers strain:

> *"It would have been nice if the therapist met my Dad … to have the therapist's viewpoint, to see for themselves."* (d2; moderate dementia; HADS 14 → 10 [12 months])



For peer review only - http://bmjopen.bmj.com/site/about/guidelines.xhtml

*"One session involving the care-recipient so they appreciate there are problems … and the effect their illness is having on spouse … might help with their self-control."* (w10; very mild dementia; HADS 16 → 16)

Two participants felt that the START sessions should have been more explicit in their exploration of the dementia future problems and prognosis:

*"More discussion of the likely course of the illness."* (s20; mild dementia; HADS 17 → 14)

*"How to prepare for what lies ahead."* (h21; moderate dementia; HADS 9 → 26)

Although the CD of relaxation techniques was popular with many respondents, others did not like it:

*"I haven't used the CD – some of which I found really irritating!"* (w22; moderate dementia; HADS 22 → 24)

*"I found the male voices off-putting on the CD – prefer all female voices."* (w3; mild young-onset dementia; HADS 19 → 8)

Appropriate time for delivery of intervention

Participants were largely recruited shortly after or at the time of dementia diagnosis. 61/75 carers ~~(81%)~~ judged that they had taken part in the START intervention at the 'right time':

*"I now feel I have all the tools before she gets worse."* (s23; very mild dementia; HADS 12 → 5)

Of those who thought the intervention should have been offered at another time, eight wanted it earlier and six later. About three quarters of carers looking after those with very mild dementia (CDR 0.5) thought the intervention was delivered at the right time, rising to over 80% of those with relatives with mild or moderate dementia (CDR 1-2). Those who wanted it later tended to have relatives with milder dementia than those asking for it earlier. Among carers who would rather have received the intervention earlier, the median time since they reported being told the diagnosis was 5.5 months, 4 months for those who were happy with the time of delivery and 1.5 months for those who would rather have received it later.



For peer review only - http://bmjopen.bmj.com/site/about/guidelines.xhtml

Respondents commented that earlier engagement with the START programme would have helped them improve their communication and thus care better or avoid making major decisions regarding social care without being equipped with the necessary knowledge of dementia:

> *"I wish I knew more, well before her condition was diagnosed, as I feel that I would have been more understanding and giving to her."* (d24; mild dementia; HADS 5 → 6)

> *"[START programme] should have started earlier before we found a live-in carer for my mother-in-law."* (d25; mild dementia; HADS 11 → 6 [12 months])

Those who felt that the intervention was delivered too early felt it would have helped them cope with their relative's later deterioration:

> *"I feel it was a little early as further down the line, I find it so much harder to cope with my mother as her Alzheimer's has got worse."* (d17; very mild dementia; HADS 8 → 14)

**Discussion**

This study is the first to qualitatively analyse dementia carers' experiences of a complex psychological intervention and thus help us to understand the mechanisms by which it is effective. We asked carers about their experiences of the therapy two years after the 8-12 week therapy began, so we were able to explore how it worked immediately and whether benefits were noted some time later.

Our main finding is that the study participants valued diverse components of the intervention. The most frequently cited aspects were relaxation techniques, education about dementia, interaction with the therapist, cognitive techniques for their own thoughts and feelings, specific strategies for behavioural management and communication techniques for the person with dementia as well as learning to seek and ask for help. Most said they continued to use techniques at two year follow-up.

The heterogeneity of responses suggests that there were no particular aspects that were commonly unnecessary or unhelpful, and that a benefit of the multicomponent nature of this therapy is that it provides a diverse menu of strategies to suit the differing circumstances of dementia carers – in terms of the relative with dementia's symptoms; carer knowledge; social situation and support from mental health and social services; preferences and coping strategies.

The HADS scores for each quoted participant allow speculation regarding the impact of their anxiety and depression levels on their mental state at the time of both the intervention and providing this feedback which is instructive in some cases. However, they are used in illustrative quotes of participants, and cannot be assumed to represent other participants holding a similar view of the intervention.



For peer review only - http://bmjopen.bmj.com/site/about/guidelines.xhtml

Some of the responses demonstrate that participants had incorrectly interpreted the intervention's messages – for example, that teaching people with dementia about their condition 'might help with their self-control' or that one should 'go along with whatever the Alzheimer's sufferer says' (the message was to avoid criticism) – reflecting the subjective experience of psychological interventions but the overall effect of the intervention was nonetheless positive. While some HADS scores went up eg the first quote from 'w1', she continued to use the relaxation techniques and felt this was beneficial; it may be that she would have felt worse without the intervention.

Suggestions about how to improve the intervention focussed on form rather than content – for example, shorter or more numerous sessions, with several carers suggesting a longer period of intervention with reduced frequency of sessions. These participants may have been experiencing higher level of psychological distress, in which case additional sessions for those who are most distressed at the end of therapy would be in line with the stepped care approach to access to psychological therapies. Alternatively, they may have benefitted most and so were reluctant to stop.

Family carers have previously reported that receiving information over a longer period helps when making decisions about care [17-19] and respondents welcomed the gradual accumulation of knowledge about dementia. Others made suggestions about broadening session attendance to include other family members or the person with dementia. While including more family members could reduce the individual feeling of therapist attention, it could broaden the impact of the intervention, and family interventions have been found to be effective in other studies.[20] Perhaps the diverse content helped the START intervention to support carers with a broad range of needs, and a flexible approach to its delivery, in terms of who is present in sessions and how they are scheduled, could assist implementation.

The contact with a professional was welcomed by many participants, who valued the empathetic approach, knowledge and interpersonal skills of the therapists. We know, from analysis of the effect of clustering by therapist, that the clinical effectiveness of the therapy was not dependent on which therapist delivered the intervention [7] so this suggests that supervised psychology graduates can deliver this therapy while maintaining a personal approach. Some carers cited a cognitive therapeutic approach as helpful and this supports research findings that cognitive reframing may be an effective aspect of individualised multicomponent interventions.[21]

*Strengths and weaknesses*

To our knowledge, our qualitative analysis of participants' experience of a clinically-effective and cost-effective psychosocial intervention aimed at improving the mental health of dementia carers is the first study of this type. In order to maximise the validity of our findings, we aimed for and succeeded in gaining, a maximum variation sample of people who completed the intervention; the participants in our study covered the spectrum of socio-demographic and clinical characteristics of a broader group of individuals who received the intervention. However, the questionnaire respondents, compared to those who did not respond, were statistically significantly younger and tended to be children rather than spouses of people with dementia, ~~younger,~~ less likely to be



For peer review only - http://bmjopen.bmj.com/site/about/guidelines.xhtml

married, more likely to be in employment rather than retired and less likely to be living with the person with dementia.

In addition to this, the respondents had reached a higher educational level than non-respondents. It may be that participants with lower literacy attainment would have had more difficulties in filling in the questionnaire. The written format also meant that we could not probe participants' answers. For example, 18 participants specified that they appreciated receiving information about dementia but we do not know the opinion of the remaining 57 participants about this. Using self-completed questionnaires, however, had the strength that the participants were free to express their views. The lack of changes after we offered participants a chance to revise their transcripts also suggests this. It also supports the idea that the START intervention had a long-lasting and consistent effect on participants; the initial questionnaire responses providing a snapshot of the participants' views but these remained constant.

There is probably some response bias, with those who valued and benefited from the therapy most or least and had the strongest feelings being more likely to respond. As we did not receive any responses from participants whose relative had severe dementia at the beginning of the intervention, we cannot make assumptions about the experience of the intervention for this group. Nonetheless, many of the respondents cared for people who progressed to severe dementia or died, so delivering the intervention early may mean that it continues to confer benefit as dementia deteriorates. We have been unable to obtain views from most carers who withdrew from the study, which would suggest that we probably under-sampled those who disliked the intervention or found it unacceptable. Nonetheless a minority of respondents did criticise the therapy, suggesting that our strategies to minimise social desirability bias were successful.

*Clinical implications and future research*

This analysis indicates aspects of the START intervention which were helpful and suggests some possible reasons for lasting clinical efficacy. Participants took part in a structured and guided intervention and their comments indicated a range of opinions about which parts were helpful. It seems that, although some individuals did not like components of the intervention, most were able to identify helpful aspects and that, by working with graduate psychologists over a period of time, they made some longer term changes. Supporting the structured sessions with a manual and CD based intervention was valuable for the two-thirds of participants who continued to use them two years later and participants commented that they also felt more capable of seeking help for future changing circumstances.

**Comment [ATS2]:** REFERENCE ADDED FOR 24 MONTH DATA:
Livingston G, Barber J, Rapaport P, et al. START (STrAtegies for RelaTives) study: A pragmatic randomised controlled trial to determine the efficacy and cost effectiveness of a manual based coping strategy programme in promoting the mental health of carers of people with dementia. *Health Technol Assess*. Forthcoming 2014.

Our analysis has implications for when best to offer the intervention to carers in order to maximise engagement and potential benefit. Responses suggest that most carers want the intervention to be delivered shortly after they had been told of a dementia diagnosis; this allows future social care planning and psychological preparation for the developing illness. Some carers for people with very mild dementia would have preferred to wait a few months following diagnosis. In clinical practice,



For peer review only - http://bmjopen.bmj.com/site/about/guidelines.xhtml

having flexibility in terms of when people are offered the START intervention could increase the acceptability of the intervention.

Overall, we found that two-thirds of carers reported continued use of the intervention after two years. This indicates a possible mechanism for continuing efficacy after the intervention's end. Retaining a copy of the manual and CD provides opportunity to revisit valued aspects of the therapy and adapt caring approaches to current challenges, while signposting to relevant voluntary organisations has been shown to be helpful[22] and empowered the participants in our study to seek help elsewhere, providing help as circumstances changed. Our findings are in line with others that multicomponent interventions are necessary in complex conditions with multiple domain pathology and that a single active ingredient may be illusory.[23, 24]

**Funding statement**

This project was funded by the National Institute for Health Research Health Technology Assessment (HTA) programme (project No 08/14/06). The authors analysed results and prepared this manuscript independently of the funding body. The views and opinions expressed therein are those of the authors and do not necessarily reflect those of the HTA programme, National Institute for Health Research, National Health Service, or the Department of Health. The study was sponsored by University College London. Neither funders nor sponsors had a role in the study design and the collection, analysis and interpretation of data and the writing of the article and the decision to submit it for publication. The researchers were independent from funders and sponsors.

**Competing interests statement**

All authors have completed the ICMJE uniform disclosure form at www.icmje.org/coi_disclosure.pdf and declare: support from Health Technology Assessment for the submitted work; no financial relationships with any organisations that might have an interest in the submitted work in the previous three years; no other relationships or activities that could appear to have influenced the submitted work.

**Ethical approval**

Ethical approval: The trial was conducted in accordance with Good Clinical Practice guidelines, the Declaration of Helsinki, the Clinical Trials Regulations and local laws and regulations. We obtained written ethics approval for the study from East London and the City Research Ethics Committee for the trial (ID: 09\H0703\84) and Research and Development permission from the local trusts. All participants gave written informed consent.



For peer review only - http://bmjopen.bmj.com/site/about/guidelines.xhtml

**Description of authors' roles**

AS, PR, CC and GL contributed to the design of the study. MM facilitated the questionnaire distribution. AS and MM analysed the data for themes and agreed on a coding frame. All authors contributed substantially to conception and design, or analysis and interpretation of data, and to drafting the article or revising it critically for intellectual content.

**Acknowledgements**

We thank Dolores Gallagher Thompson for her original manual and allowing us to adapt it; the participating carers; the Camden and Islington NHS Foundation Trust, University College London Hospital, the North East London Foundation Trust, and the North Essex Partnership Foundation; Vincent Kirchner and Lisa Gee for referring many patients; members of the steering committee and the data monitoring committee. The START research team acknowledges the support of the National Institute for Health Research, through the Dementia and Neurodegenerative Research Network (DeNDRoN).

For peer review only



For peer review only - http://bmjopen.bmj.com/site/about/guidelines.xhtml

## References

1 Alzheimer's Society. (2012). Dementia 2012: A national challenge.

2 Mahoney R, Regan C, Katona C, et al. Anxiety and depression in family caregivers of people with Alzheimer Disease: the LASER-AD study. *Am J Geriatr Psychiatry* 2005;13(9):795-801. doi: 10.1097/00019442-200509000-00008

3 Gallagher D, Ni Mhaolain A, Crosby L, et al. Determinants of the desire to institutionalize in Alzheimer's caregivers. *Am J Alzheimers Dis Other Demen* 2011;26:205-211. doi: 10.1177/1533317511400307

4 Pinquart M, Sörensen S. Helping caregivers of persons with dementia: which interventions work and how large are their effects? *International Psychogeriatrics* 2006;18(4):577-95. doi: 10.1017/S1041610206003462

5 Talking therapies: a 4 year plan of action [Internet]. Department of Health; 2011. Available from: https://www.gov.uk/government/uploads/system/uploads/attachment_data/file/213765/dh_123985.pdf

6 Gallagher D, Thompson L, Silven D, et al. Problem solving for caregivers: Class leaders' manual. VA Palo Alto Health Care System and Stanford University, Palo Alto, CA. 1985.

7 Livingston G, Barber J, Rapaport P, et al. Clinical effectiveness of a manual based coping strategy programme (START, STrAtegies for RelaTives) in promoting the mental health of carers of family members with dementia: pragmatic randomised controlled trial. *BMJ* 2013;347:f6276. doi: 10.1136/bmj.f6276

8 Knapp M,King D, Romeo R, et al. Cost effectiveness of a manual based coping strategy programme in promoting the mental health of family carers of people with dementia (the START (STrAtegies for RelaTives) study): a pragmatic randomised controlled trial. *BMJ* 2013;347:f6342. doi: http://dx.doi.org/10.1136/bmj.f6342

9 Wells M, Williams B, Treweek S, et al. Intervention description is not enough: evidence from an in-depth multiple case study on the untold role and impact of context in randomised controlled trials of seven complex interventions. *Trials* 2012;13:95. doi: 10.1186/1745-6215-13-95

10 Li R, Cooper C, Livingston G. Relationship of coping style to mood and anxiety disorders in dementia carers. *Curr Opin Psychiatry* 2014;27(1):52-6. doi: 10.1097/YCO.0000000000000020

11 Murphy R, Cooper Z, Hollon S, et al. How do psychological treatments work? Investigating mediators of change. *Behav Res Ther* 2009;47(1):1-5. doi: 10.1016/j.brat.2008.10.001

12 Hoddinott P, Craig L, MacLennan G, et al. Process evaluation for the Feeding Support Team (FEST) randomised controlled feasibility trial of proactive and reactive telephone support for breastfeeding



For peer review only

For peer review only - http://bmjopen.bmj.com/site/about/guidelines.xhtml

women living in disadvantaged areas. *BMJ Open* 2012;2:e001039. doi: 10.1136/bmjopen-2012-001039

13 Tonkin-Crine S, Bishop F, Ellis M, et al. Exploring patients' views of a cognitive behavioral therapy-based website for the self-management of irritable bowel syndrome symptoms. *J Med Internet Res* 2013;15(9):e190. doi: 10.2196/jmir.2672

14 Penn L, Moffatt S, White M. Participants' perspective on maintaining behaviour change: a qualitative study within the European Diabetes Prevention Study. *BMC Public Health* 2008;8:235. doi: 10.1186/1471-2458-8-235

15 Zigmond A, Snaith R. The hospital anxiety and depression scale. *Acta Psychiatr Scand* 1983;67:361-70. doi: 10.1111/j.1600-0447.1983.tb09716.x

16 Hughes C, Berg L, Danziger W, et al. A new clinical scale for the staging of dementia. *Br J Psychiatry* 1982;140:566-72. doi: 10.1192/bjp.140.6.566

17 Wald C, Fahy M, Walker Z, et al. What to tell dementia caregivers—the rule of threes. *Int J Geriatr Psychiatry* 2003;18:313-7. doi: 10.1002/gps.828

18 Hirschman K, Kapo J, Karlawish J. Why doesn’t a family member of a person with advanced dementia use a substituted judgment when making a decision for that person? *Am J Geriatr Psychiatry* 2006;14:659-67. doi: 10.1097/01.JGP.0000203179.94036.69

19 Livingston G, Leavey G, Manela M, et al. Making decisions for people with dementia who lack capacity: qualitative study of family carers in UK. *BMJ* 2010;341:c4184. doi: 10.1136/bmj.c4184

20 Marriott A, Donaldson C, Tarrier N, et al. Effectiveness of cognitive-behavioural family intervention in reducing the burden of care in carers of patients with Alzheimer's disease. *Br J Psychiatry* 2000;176:557-62. doi: 10.1192/bjp.176.6.557

21 Vernooij-Dassen M, Draskovic I, McCleery, et al. Cognitive reframing for carers of people with dementia. *Cochrane Database of Systematic Reviews* 2001;11:CD005318. doi: 10.1002/14651858.CD005318.pub2

22 Sörensen S, Duberstein P, Gill D, et al. Dementia care: mental health effects, intervention strategies and clinical implications. *Lancet Neurol* 2006;5:961-73. doi: 10.1016/S1474-4422(06)705993

23 Spijker A, Vernooij-Dassen M, Vasse E, et al. Effectiveness of nonpharmacological interventions in delaying the institutionalization of patients with dementia: A meta-analysis. *J Am Geriatr Soc* 2008;56:1116-1128. doi: 10.1111/j.1532-5415.2008.01705.x

24 Olazarán J, Reisberg B, Clare L, *et al*. Nonpharmacological therapies in Alzheimer's disease: a systematic review of efficacy. *Dement Geriatr Cogn Disord* 2010;30:161-178. doi: 10.1159/000316119



For peer review only

For peer review only - http://bmjopen.bmj.com/site/about/guidelines.xhtml

**Table 1.** **Baseline carer characteristics of questionnaire respondents and non-respondents**

| | | Respondents (n=75) Mean (SD) | Non-respondents (n=98) Mean (SD) |
|---|---|---|---|
| **Age** | | 59.3 (13.7) Range: 18 - 85 | 64.1 (15.1) Range: 19 - 88 |
| | | | |
| | **Characteristic** | **n (%) of respondents (n=75)** | **n (%) of non-respondents (n=98)** |
| **Gender** | Female | 49 (65.3) | 67 (68.4) |
| | | | |
| **Ethnicity** | White UK | 58 (78.4) | 67 (68.4) |
| | White other | 4 (5.4) | 12 (12.2) |
| | Black and minority ethnic | 12 (16.2) | 19 (19.4) |
| | Missing | 1 | 0 |
| | | | |
| **Marital status** | Married / common law | 42 (56.0) | 63 (64.3) |
| | | | |
| **Education** | No qualifications | 14 (18.7) | 31 (31.6) |
| | School level | 24 (32.0) | 27 (27.6) |
| | Further education | 23 (30.7) | 24 (24.5) |
| | Other | 14 (18.7) | 16 (16.3) |
| | | | |
| **Employment** | Full time | 17 (22.7) | 19 (19.4) |
| | Part time | 17 (22.7) | 10 (10.2) |
| | Retired | 29 (38.7) | 51 (52.0) |
| | Not working | 12 (16.0) | 18 (18.4) |
| | | | |
| **Relationship to patient** | Spouse / partner | 31 (41.3) | 47 (48.0) |
| | Child | 34 (45.3) | 37 (37.8) |
| | Other | 10 (13.3) | 14 (14.3) |
| | | | |
| **Living with patient?** | Yes | 44 (58.7) | 69 (70.4) |



For peer review only - http://bmjopen.bmj.com/site/about/guidelines.xhtml

**Table 2.** **Clinical characteristics of questionnaire respondents and non-respondents**

| | | Respondents (n=75) Median (SD) | Non-respondents(n=98) Median (SD) |
|---|---|---|---|
| | | | |
| **Number of months since initial diagnosis** | | 3.5 (19.8) Range: 0 – 96 | 4.0 (17.3) Range: 0 – 108 |
| | | | |
| | | **Respondents (n=75) Mean (SD)** | **Non-respondents(n=98) Mean (SD)** |
| | | | |
| **HADS Baseline** | | 13.6 (6.9) | 13.4 (7.7) |
| | | | |
| **HADS 24m** | | 14.2 (8.1) | 12.9 (8.3) |
| | | | |
| | **Characteristic** | **n (%) of respondents (n=75)** | **n (%) of non-respondents (n=98)** |
| | | | |
| **CDR BL** | Very mild | 15 (20.0) | 15 (15.3) |
| | Mild | 41 (54.7) | 50 (51.0) |
| | Moderate | 19 (25.3) | 29 (29.6) |
| | Severe | 0 | 2 (2.0) |
| | Missing | 0 | 2 (2.0) |
| | | | |
| **CDR 24m** | Mild | 21 (30.4) | 15 (31.3) |
| | Moderate | 26 (37.7) | 19 (39.6) |
| | Severe | 11 (15.9) | 6 (12.5) |
| | Care recipient died | 11 (15.9) | 8 (16.7) |
| | Missing | 6 | 9 |
| | Withdrawn | 0 | 41 |

**Legend: HADS: Hospital Anxiety and Depression score, CDR: Clinical Dementia Rating score**



For peer review only

**Figure 1~~3~~. Aspects of START intervention which were frequently described as helpful by participants**

| |
|---|
| **Influences on carer attitude**<br>• Greater acceptance of diagnosis and situation<br>• More tolerant to person with dementia<br>• Validation of own feelings |
| **Specific components of START intervention**<br>• CD and relaxation exercises<br>• Behavioural and communication advice<br>• Education about dementia |
| **Interaction with therapist**<br>• Sharing concerns with an independent person<br>• Empathy from therapist<br>• Reframing of negative automatic thoughts |
| **Continued use of techniques and attitudes**<br>• Seeking similar approaches through voluntary organisations<br>• Sharing techniques with friends and relatives |

For peer review only



For peer review only - http://bmjopen.bmj.com/site/about/guidelines.xhtml

**Influences on carer attitude**

- Greater acceptance of diagnosis and situation
- More tolerant to person with dementia
- Validation of own feelings

**Specific components of START intervention**

- CD and relaxation exercises
- Behavioural and communication advice
- Education about dementia

**Interaction with therapist**

- Sharing concerns with an independent person
- Empathy from therapist
- Reframing of negative automatic thoughts

**Continued use of techniques and attitudes**

- Seeking similar approaches through voluntary organisations
- Sharing techniques with friends and relatives

90x63mm (300 x 300 DPI)

view only
